# A pipeline for copy number profiling of single circulating tumour cells to assess intrapatient tumour heterogeneity

**DOI:** 10.1002/1878-0261.13174

**Published:** 2022-07-08

**Authors:** Teoman Deger, Pauline A. J. Mendelaar, Jaco Kraan, Wendy J. C. Prager‐van der Smissen, Michelle van der Vlugt‐Daane, Eric M. J. Bindels, Anieta M. Sieuwerts, Stefan Sleijfer, Saskia M. Wilting, Antoinette Hollestelle, John W. M. Martens

**Affiliations:** ^1^ Department of Medical Oncology Erasmus MC Cancer Institute Rotterdam The Netherlands; ^2^ Department of Hematology Erasmus MC Cancer Institute Rotterdam The Netherlands

**Keywords:** bioinformatics pipeline, breast cancer, circulating tumour cells, next‐generation sequencing, prostate cancer, single‐cell genomics, whole genome amplification

## Abstract

Intrapatient tumour heterogeneity is likely a major determinant of clinical outcome in cancer patients. To assess heterogeneity in a minimally invasive manner, methods to perform single circulating tumour cell (CTC) genomics at high resolution are necessary. However, due to the rarity of CTCs, development of such methods is challenging. Here, we developed a modular single CTC analysis pipeline to assess intrapatient heterogeneity by copy number (CN) profiling. To optimize this pipeline, spike‐in experiments using MCF‐7 breast cancer cells were performed. The VyCAP puncher system was used to isolate single cells. The quality of whole genome amplification (WGA) products generated by REPLI‐g and *Ampli1*™ methods, as well as the results from the Illumina Truseq and the *Ampli*1™ LowPass library preparation techniques, was compared. Moreover, a bioinformatic pipeline was designed to generate CN profiles from single CTCs. The optimal combination of *Ampli*1™ WGA and Illumina Truseq library preparation was successfully validated on patient‐derived CTCs. In conclusion, we developed a novel modular pipeline to isolate single CTCs and subsequently generate detailed patient‐derived CN profiles that allow assessment of intrapatient heterogeneity in future studies.

## Graphical abstract

Here, we present a technical study on the development of a single circulating tumour cell (CTC) analysis pipeline to assess intrapatient heterogeneity by copy number profiling. We compared two whole genome amplification methods, two library preparation methods and three quality control assays for single‐cell analysis after CellSearch enrichment and VyCAP punching. The established modular pipeline was applied to blood‐derived single cells from patients with metastatic breast or prostate cancer.

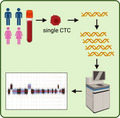

**Abbreviations**
APC: allophycocyan
BC: breast cancer
CK: cytokeratin
CN: copy number
CNV: copy number variation
CTC(s): circulating tumour cell(s)
DAPI: 4,2‐diamidino‐2‐phenylindole dihydrochloride
FACS: fluorescence‐activated cell sorting
FDA: Food and Drug Administration
FITC: fluorescein isothiocyanate
HBD: healthy blood donor
MAD: median absolute deviation
PC: prostate cancer
PE: phycoerythrin
QC: quality control
RR: recovery rate
SR: success rate
WGA: whole genome amplification

## 1 Introduction

Approximately 90% of cancer deaths are due to metastatic disease that has acquired resistance to currently available treatments [1, 2]. Therefore, elucidating the pathophysiology of metastasis and treatment failure is pivotal. Molecular tumour heterogeneity is increasingly recognized as causal [3, 4, 5, 6] and, as a result, development of methods that allow us to capture intrapatient heterogeneity, preferably repeatedly, in a patient‐friendly manner is imperative. Although tissue biopsies are the conventional method to assess the molecular characteristics of metastases, taking a biopsy is a cumbersome procedure and not always possible due to inaccessibility of target lesions. Apart from technical challenges, a single biopsy may not be representative with regard to sampling intratumour heterogeneity within a patient due to spatial and temporal tumour evolution [3, 7, 8]. In patients with metastatic disease, circulating tumour cells (CTCs) present in the peripheral blood can be derived from both primary and metastatic lesions [9]. Additionally, intrapatient metastatic lesions are able to progress distinctively [10]. Therefore, the analysis of intact single CTCs potentiates the assessment of intrapatient heterogeneity. As CTCs can be acquired through minimally invasive peripheral blood draws, they can be taken consecutively to monitor molecular changes during disease progression and, importantly, under treatment pressure. The molecular analysis of CTCs through these ‘liquid biopsies’ is therefore increasingly suggested as a tool to improve precision medicine in the future [11, 12, 13, 14].

Although multiple methods for the analysis of single cell have been developed, characterization of single CTCs is currently not yet routinely applied. The isolation of CTCs is mostly based on the FDA‐approved CellSearch method®, the current gold standard for CTC identification and enumeration (Menarini Silicon Biosystems, Inc), which has proven to be a reliable prognostic tool in multiple metastatic cancer types [15, 16]. Subsequent to CellSearch enrichment, pure CTCs can be isolated through different methods, including fluorescence‐activated cell sorting (FACS), micromanipulation using electric cages (i.e. DEParray) and microwell‐punching (i.e. VyCAP) [17, 18]. However, none of the developed methods for single CTC analysis has been implemented into daily clinical practice due to their respective shortcomings. Technical difficulties include incompatibility with fixed whole blood samples and quantity of the input material, precluding high‐throughput single‐cell analysis methods such as the Fluidigm and 10× Genomics systems [19]. Since most of these methods are designed to be applied to fresh frozen tissue, they are not optimized for the analysis of the limited number of single cells obtained through liquid biopsies as this demands a high accuracy of both the applied isolation method and the used whole genome amplification (WGA) method [20]. In addition, although current bioinformatic methods to analyse the acquired single‐cell genomic data mostly allow for genomic variant detection, the analysis is often restricted to predefined algorithms [21] which can lead to a selection bias regarding the results.

To enable copy number variation (CNV) profiling of single CTCs, we here report the development of a modular pipeline with multiple entry points in regard to the enrichment method, isolation method and analysis method. We demonstrate that the combined use of CellSearch enrichment and VyCAP punching enables the isolation of pure, single CTCs in small volumes and characterization of CTCs from patient‐derived blood samples. In addition, we developed a new bioinformatic pipeline combining a number of published and accessible R packages with a limited hands‐on time which can be routinely applied.

## 2 Materials and methods

### 2.1 Cell line, healthy blood donors and patients

MCF‐7 breast cancer cells (ATCC, HTB‐22; authenticated by Short Tandem Repeat profiling) were cultured in RPMI1640 GlutaMax medium (Thermo Fisher Scientific, Waltham, MA) supplemented with 10% heat‐inactivated fetal bovine serum, 100 µg·mL^−1^ penicillin and 80 µg·mL^−1^ streptomycin. Seven healthy blood donors (HBDs) donated 10 to 50 mL of blood, which was used for the spike‐in experiments and to isolate leukocytes to generate a copy number (CN) reference panel. Finally, we used blood samples from four cancer patients: two female patients with metastatic breast cancer (BC) and two male patients with metastatic prostate cancer (PC). These patients participated in various clinical studies designed in accordance with the standards set by the Declaration of Helsinki and approved by the Medical Ethics Committee of the Erasmus University Medical Center (MEC 17‐238, MEC 16‐703 and MEC 16‐449). All patients and donors gave written informed consent. Blood (8–10 mL) was drawn in CellSave collection tubes (Menarini Silicon Biosystems, Castel Maggiore (BO), Italy) which contain EDTA and a cell‐stabilizing fixative. Blood samples were kept on room temperature and were processed within 96 h of collection to avoid degradation of target cells [22].

### 2.2 Spike‐in experiments

MCF‐7 cells were harvested and suspended in phosphate‐buffered saline (PBS, Thermo Fisher Scientific) and counted using a LSRFortessa flow cytometer (BD Biosciences, Franklin Lakes, NJ). To obtain a reliable cell count, the cell suspension was counted until a minimum of 10 000 events, of which 1000 fluorescent counting beads (Beckman Coulter, Indianapolis, IN) were acquired. Afterwards, 500 MCF‐7 cells were spiked into 7.5 mL of HBD blood.

### 2.3 CellSearch‐based cell staining and enrichment

To collect CTCs or (spiked) cells from blood (1 CTC per 10^6‐7^ peripheral blood mononuclear cells on average [23]), target cells (MCF‐7 cells, patient CTCs or HBD leukocytes) were enriched and stained by the CellSearch method (Menarini Silicon Biosystems) as previously reported [24]. The CellSearch Circulating Tumor Cell Kit was used for the enrichment of spiked‐in MCF‐7 cells and patient‐derived CTCs. MCF‐7 cells or CTCs were enriched using anti‐epithelial cell adhesion molecule (EpCAM) antibodies coupled to paramagnetic nanoparticles. To enrich leukocytes, we used anti‐melanoma cell adhesion molecule (MCAM/CD146) antibodies for which a subset of T‐lymphocytes is positive [25]. The CellSearch method yields an enriched suspension of 350 µL when captured in a CellSearch MagNest with an average CTC to leukocyte ratio of approximately 1 in 10^3^ cells [26]. In addition, we used the CellSearch CX‐9 protocol which encompasses the same steps as the enumeration protocol excluding the transmission of the sample to a MagNest and results in an enriched cell fraction of 950 µL. Target cells transferred to the MagNest were enumerated on the CellTracks analyser system by a certified technician.

### 2.4 Single‐cell isolation using the VyCAP puncher system

For the isolation of single cells, the VyCAP puncher system (Enschede, The Netherlands) was used as described by the manufacturer [17]. The VyCAP system uses self‐seeding microwell chips which contain 6400 70 µm microwells with each bottom containing a single 5‐µm pore. Under vacuum, a fluid containing the target cells, in our case the 950 µL CellSearch‐enriched CX‐9 fraction, can be used to load the chip. Cells are guided into separate wells by laminar flow, that is diverted after a captured cell blocks the pore of that well, ultimately resulting in individually separated cells per well. After loading, the microchip is imaged at four fluorescence channels (DAPI, fluorescein isothiocyanate (FITC), PE and APC) with the Nikon Eclipse Ti fluorescence microscope (Nikon Instruments, Tokyo, Japan). All immunofluorescent signals are automatically analysed, and wells which contain a potential leukocyte (DAPI^+^/CK^‐^/CD45^+^) or a CTC or an MCF‐7 cell (DAPI^+^/CK^+^/CD45^‐^) are automatically presented and manually selected for punching after verification. The bottom of the well containing the selected cell is punched out of the microwell chip by a precision needle and captured in the cap of a 200‐µL tube. Highest recovery was obtained with cells isolated in 35 µL mineral oil in the caps of dome‐capped tubes (https://www.vycap.com/inhoud/uploads/VyCAP‐Puncher‐optimized‐mineral‐oil‐Ampli1‐WGA‐protocol‐1.1.pdf).

### 2.5 Whole genome amplification of punched cells

**Fig. 1:**
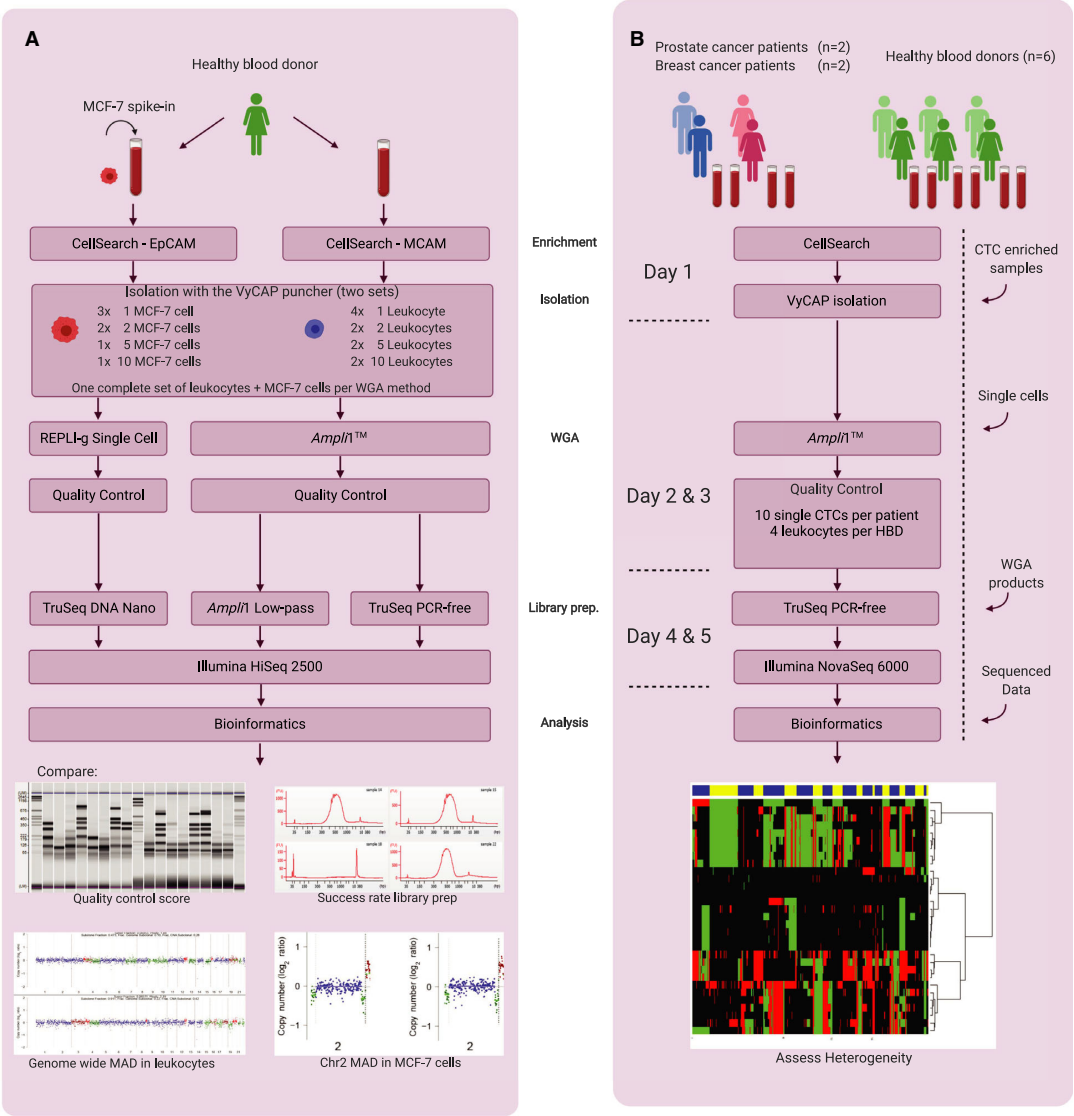
Schematic workflow of pipeline optimization and validation on clinical samples. (A) Workflow for determining the optimal WGA method and library preparation method. (B) Validation of the optimal pipeline to obtain a single‐cell control panel from HBDs and CN profiles from single CTCs. Workflow in days is indicated on the left. The arrows on the right represent entry points after alternative methods. MAD, median absolute deviation.

An experiment was designed to determine which WGA method performed better to amplify genomes from single CellSearch‐enriched, VyCAP‐punched cells (Fig. 1A). Two tubes of 7.5 mL HBD blood were used for this purpose. MCF‐7 cells were spiked‐in the first tube while the second tube was used to provide HBD leukocytes. Subsequently, two sets of leukocytes (i.e. 4 × 1, 2 × 2, 2 × 5 and 2 × 10 cells per tube cap) and spiked‐in MCF‐7 cells (i.e. 3 × 1, 2 × 2, 1 × 5 and 1 × 10) were isolated using the VyCAP puncher (Table 1, samples 1‐17). The first complete set (MCF‐7 cells and leukocytes) was subjected to the *Ampli*1™ WGA Kit (Menarini Silicon Biosystems) according to manufacturer’s recommendations. *Ampli*1™ WGA is a PCR‐based amplification method after *MseI* digestion of the genomic DNA using an adaptor with a universal primer. The second complete set of punched cells was subjected to the REPLI‐g Single‐Cell WGA Kit (Qiagen, Hilden, Germany) using manufacturer’s recommendations and 16 h of amplification. REPLI‐g WGA uses multiple displacement linear amplification of the genomic DNA using phi29 polymerase. Reagents were added against the tube wall and centrifuged briefly (i.e. 12 000 rcf for 1 min) to collect the reaction mixture underneath the mineral oil. Amplified samples were stored at −20 °C up to 1 month.

**Table 1:** Overview of samples used in the optimization of the WGA and sequencing pipeline. Samples are grouped by: (Top) HBD leukocyte samples, (Middle) MCF‐7 spike‐in samples and (Bottom) additional controls. VyCAP QC: scores can range from QC‐0 to QC‐7 for WGA samples. QC‐8 can be obtained in undigested (genomic) DNA only. *Illumina TruSeq versus *Ampli*1 LowPass library prep yield Wilcoxon signed rank *P* = 7.15^‐7^. No, number; n.a., not available.

Sample No.	Sample Name	Sample Type	Donor/Source	Gender	Input	VyCAP QC score	Yield TruSeq (nm)*	Yield LowPass (nm)*
1	Leuko, 1A	Leukocyte	HBD0	Female	1 cell	7	4.7	45.8
2	Leuko, 1B	Leukocyte	HBD0	Female	1 cell	7	3.0	19.2
3	Leuko, 1C	Leukocyte	HBD0	Female	1 cell	7	5.3	82.8
4	Leuko, 1D	Leukocyte	HBD0	Female	1 cell	7	6.9	123.1
5	Leuko, 2A	Leukocyte	HBD0	Female	2 cells	7	5.3	53.2
6	Leuko, 2B	Leukocyte	HBD0	Female	2 cells	7	7.9	47.0
7	Leuko, 5A	Leukocyte	HBD0	Female	5 cells	7	8.3	75.1
8	Leuko, 5B	Leukocyte	HBD0	Female	5 cells	7	4.9	83.9
9	Leuko, 10A	Leukocyte	HBD0	Female	10 cells	7	4.0	23.1
10	Leuko, 10B	Leukocyte	HBD0	Female	10 cells	7	3.5	7.3
11	MCF‐7, 1A	Tumour cell	MCF‐7		1 cell	7	3.6	22.4
12	MCF‐7, 1B	Tumour cell	MCF‐7		1 cell	7	4.9	7.0
13	MCF‐7, 1C	Tumour cell	MCF‐7		1 cell	7	4.9	101.3
14	MCF‐7, 2A	Tumour cell	MCF‐7		2 cells	7	4.3	82.3
15	MCF‐7, 2B	Tumour cell	MCF‐7		2 cells	7	2.4	65.5
16	MCF‐7, 5	Tumour cell	MCF‐7		5 cells	7	6.6	4.8
17	MCF‐7, 10	Tumour cell	MCF‐7		10 cells	7	5.1	24.7
18	Leuko, QC‐6	Leukocyte	HBD0	Female	1 cell	6	8.1	55.3
19	MCF‐7, QC‐6	Tumour cell	MCF‐7		1 cell	6	1.9	80.2
20	MCF‐7, QC‐5	Tumour cell	MCF‐7		1 cell	5	1.4	25.9
21	VyCAP Ctrl 1	Tumour cell	MCF‐7, VyCAP		1 cell	7	4.3	5.8
22	VyCAP Ctrl 2	Tumour cell	MCF‐7, VyCAP		1 cell	7	3.4	93.7
23	MCF‐7, *Ampli*1	Tumour DNA	MCF‐7		1 ng DNA	7	1.4	15.5
24	MCF‐7, Genomic	Tumour DNA	MCF‐7		2.5 µg DNA	8	0.2	n.a.

### 2.6 Quality control of WGA products

The quality of the REPLI‐g and *Ampli*1^TM^ WGA products was assessed with three different methods. First, we used a VyCAP‐developed multiplex PCR [27] which we modified. The modified multiplex PCR generates eight amplicons, all from different chromosome arms. All amplicons except one are designed between *MseI* sites. The *MseI*‐containing amplicon is included to differentiate between undigested (genomic) DNA and digested WGA products. The modification included omitting the PCR primer pairs for the amplicons of 606 bp, containing two internal *MseI* sites, and 1009 bp, of which the target fragment is too long for *Ampli*1™ amplification. Both amplicons are only present in samples with intact genomic DNA and therefore do not contribute to the remaining multiplex on single cells. The multiplex PCR was performed with the QIAGEN Multiplex PCR plus Kit according to manufacturer’s recommendations. We compared our modified VyCAP quality control (QC) with the four‐amplicon *Ampli1*™ QC Kit (Menarini Silicon Biosystems), performed according to manufacturer’s instructions. PCR products were visualized on the MultiNA Microchip Electrophoresis system (Shimadzu, Kyoto, Japan). A QC score for each WGA product was established by counting the number of PCR bands generated by the two multiplex PCR QC methods (i.e. QC‐0 to QC‐7 for modified VyCAP and QC‐0 to QC‐4 for *Ampli1*™).

Finally, we used the LINE‐1 directed mFAST‐SeqS method described by Belic et al. [28] on 1 ng of *Ampli1*™ WGA product in a subset of 24 samples and 12 HBD‐derived leukocytes. This method counts the amount of sequence reads of LINE‐1 elements per chromosome arm. The reads of the 24 samples were normalized per chromosome using the total mapped reads per sample and divided by the average normalized reads of the 12 leukocytes to get distribution ratios per chromosome arm. The distribution ratios per HBD leukocyte were obtained by dividing by the averages of the other 11 leukocytes. In addition to the exclusion of the chromosome arms 13p, 14p, 15p, 21p, 22p, and Yp and Yq as described by Belic et al. we also excluded Xp and Xq as leukocytes were derived from both male and female HBDs.

### 2.7 Preparation of sequencing libraries

We compared the *Ampli*1™ LowPass Kit (Menarini Silicon Biosystems) with the TruSeq PCR‐free Library Preparation Kit (Illumina, San Diego, CA). For this purpose, sequencing libraries from *Ampli*1™ WGA products of all samples with QC‐7 generated from the two punching sets as described in Fig. 1A were generated. Additional control samples included two positive controls with *Ampli*1™ WGA products that had generated high‐quality sequencing libraries before provided by VyCAP, a WGA product of a single leukocyte with a QC‐6, the WGA products of two single MCF‐7 cells with QC‐5 and QC‐6, MCF‐7 DNA (1 ng, equivalent to ~ 200 cells) amplified with the *Ampli*1™ WGA Kit and *MseI*‐digested genomic MCF‐7 DNA (2.5 µg, not subjected to WGA) (Table 1, samples 18–24). A sequencing library for the last sample could not be prepared using *Ampli*1™ LowPass library preparation due to the absence of compatible adaptor sequences and was only sequenced after TruSeq PCR‐free preparation. The *Ampli1*™ LowPass library preparation was performed according to the manufacturer’s protocol. This kit builds on the amplification adaptors attached during *Ampli*1™ WGA and therefore requires less hands‐on time compared with the TruSeq kit. Furthermore, this kit includes an additional PCR amplification and yields libraries with high concentration. The TruSeq PCR‐free kit requires 2 µg adaptor‐free WGA product as input material. *Ampli*1™ WGA products are therefore not directly compatible with the TruSeq PCR‐free kit and require additional conversions. First, higher concentrations of double‐stranded WGA product using the *Ampli*1™ ReAMP/ds kit (Menarini Silicon Biosystems) were acquired. Second, the adaptor sequences were enzymatically removed (20 µL WGA product, 50 U *MseI*, 3 h at 37 °C; New England Biolabs, Ipswich, MA). Finally, the WGA products were purified with SPRI beads (Beckman Coulter) to obtain a product compatible with the TruSeq PCR‐free kit. The fragment sizes of the libraries were measured by the High Sensitivity DNA kit on the Bioanalyzer 2100 platform (Agilent, Santa Clara, CA). Library concentrations were determined according to the NEBNext Library Quant Kit (New England Biolabs). Libraries with a minimum concentration of 2 nm and a median fragment size of 700 bp were pooled for sequencing.

### 2.8 Acquiring patient‐derived CTCs

The two metastatic breast cancer patients donated 2 tubes of blood of which one was used for standard CellSearch CTC enumeration and one was enriched using the CellSearch CX‐9 protocol after which single CTCs were isolated using the VyCAP system. The two metastatic prostate cancer patients donated 1 tube of blood which was processed through the standard CellSearch enumeration method after which the cell fraction was collected from the MagNest and CTCs were isolated using the VyCAP method. All patients had a CTC count of > 100 CTCs per 7.5 mL blood.

### 2.9 Sequencing and computational analyses

MCF‐7 cells from spike‐in experiments were sequenced on the HiSeq 2500 system (Illumina) for 50 bp single‐ended reads aimed for 10 million (10 M) reads per sample. Patient‐derived CTCs were sequenced on the NovaSeq 6000 system (Illumina) for 100 bp paired‐end aimed for 10 M reads per sample. FASTQ files were mapped using Burrows‐Wheeler Alignment for maximal exact matches (BWA‐MEM) v0.7.17 to the human reference genome hg19 using default settings [29]. Mapped reads were sorted and indexed using Samtools v1.7 [30]. The data were preprocessed with the QDNAseq R package v1.22.0 [31] after which the sequence reads in nonoverlapping regions of a preset length (bins) were counted using the QDNAseq script. Furthermore, this script removes low‐quality reads, normalizes the remaining reads, corrects for GC‐content and mappability and excludes reads mapped to the ENCODE’s Blacklist Regions. Analysis was performed on bins of 15 kb, 100 kb, 500 kb and 1 Mb lengths. For each bin size, the variance (given as σ̂_Δ_) and the number of nonassigned (i.e. empty) bins were examined. At 100‐kb resolution, most data (24 579 out of 30 970 total bins) were retained with a low σ̂_Δ_. In total, 6391 bins were blacklisted as either telomeric or centromeric regions, poorly mappable by GC content or similar regions. The excluded regions are given in Table S1. A higher percentage of bins are retained at larger bin sizes, however (e.g. 500 kb and up), with the trade‐off of lower resolution. The QDNAseq readcount data of leukocytes (*n* = 13) from six HBDs (Table 2, checked samples) were used as normal control input for the NoWaves R package v0.7 using default settings [32]. NoWaves implements normal control data from a recommended minimum of six control samples to correct read counts of CTCs for biases introduced in the pipeline (e.g. amplification bias and sequencing bias). Subsequently, the CGHcall R package v2.48.0 [33] was used to segment the data through the DNAcopy algorithm [34]. CGHcall provides additional settings to optimally call CNVs from single cells in which gains and losses of chromosomal regions are limited to CN changes in integral values. We calibrated the settings of CGHcall to call a minimum of CNVs in the HBD calibration set, while still calling known MCF‐7 CN aberrations from genomic DNA correctly, using the following settings: undo.SD = 4, clen = 10 [35]. For optimal single‐cell CNV calling, which was confirmed with a profile from a single MCF‐7 cell, the relSDlong = 2 and cellularity = 1 settings were used. Finally, we applied unsupervised hierarchical clustering using the WECCA R package v0.41 to the CNV‐called data [36]. The complete end‐to‐end code for R‐studio can be found in the Data S1.

**Table 2:** Overview of *Ampli*1‐amplified HBD samples obtained to calibrate the bioinformatic pipeline. Samples are grouped per donor. Checkmarks indicate HBD leukocytes used in the calibration set. VyCAP QC: scores can range from QC‐0 to QC‐7. Ampli1 QC: scores can range from QC‐0 to QC‐4. No, number.

Sample No.	Sample Name	Sample Type	Donor/Source	Gender	Input	VyCAP QC score	Ampli1 QC score	Yield TruSeq (nm)	Used in calibration set
25	HBD1‐1	Leukocyte	HBD1	Female	1 cell	7	2	2.8	✔
26	HBD1‐2	Leukocyte	HBD1	Female	1 cell	6	3	3.8	
27	HBD1‐3	Leukocyte	HBD1	Female	1 cell	6	2	2.0	
28	HBD1‐4	Leukocyte	HBD1	Female	1 cell	7	4	3.3	
29	HBD2‐1	Leukocyte	HBD2	Male	1 cell	7	4	2.9	✔
30	HBD2‐2	Leukocyte	HBD2	Male	1 cell	7	4	3.4	
31	HBD2‐3	Leukocyte	HBD2	Male	1 cell	7	4	4.6	
32	HBD2‐4	Leukocyte	HBD2	Male	1 cell	7	4	3.4	✔
33	HBD3‐1	Leukocyte	HBD3	Female	1 cell	7	4	3.9	✔
34	HBD3‐2	Leukocyte	HBD3	Female	1 cell	7	4	3.5	
35	HBD3‐3	Leukocyte	HBD3	Female	1 cell	5	3	2.1	
36	HBD3‐4	Leukocyte	HBD3	Female	1 cell	7	4	2.2	✔
37	HBD4‐1	Leukocyte	HBD4	Male	1 cell	7	1	8.6	
38	HBD4‐2	Leukocyte	HBD4	Male	1 cell	7	3	6.4	✔
39	HBD4‐3	Leukocyte	HBD4	Male	1 cell	7	4	2.9	✔
40	HBD4‐4	Leukocyte	HBD4	Male	1 cell	7	4	5.8	✔
41	HBD5‐1	Leukocyte	HBD5	Male	1 cell	7	2	7.5	✔
42	HBD5‐2	Leukocyte	HBD5	Male	1 cell	7	3	10.2	✔
43	HBD5‐3	Leukocyte	HBD5	Male	1 cell	7	4	10.9	
44	HBD5‐4	Leukocyte	HBD5	Male	1 cell	7	4	9.9	
45	HBD6‐1	Leukocyte	HBD6	Female	1 cell	6	4	6.6	✔
46	HBD6‐2	Leukocyte	HBD6	Female	1 cell	7	3	10.0	✔
47	HBD6‐3	Leukocyte	HBD6	Female	1 cell	7	4	5.8	
48	HBD6‐4	Leukocyte	HBD6	Female	1 cell	6	4	10.5	✔

## 3 Results

### 3.1 *Ampli*1™ versus REPLI‐g WGA on VyCAP‐isolated CTCs

We first determined the best‐suited WGA method for generating CNV profiles from CellSearch‐fixed single CTCs (Fig. 1A). Therefore, WGA was performed on two sets of punched MCF‐7 cells and leukocytes using two WGA methods: the *Ampli*1™ WGA method and the REPLI‐g WGA method. The quality of the WGA products was measured using the modified VyCAP WGA QC multiplex PCR. QC scores varied from zero bands in the negative control (QC‐0, Fig. S1, lane D) to a maximum score of eight bands for the genomic DNA positive control sample (QC‐8, Fig. S1, lane B). For WGA products, only seven bands could be maximally generated (QC‐7, Fig. S1, lane C). This is because the primers generating the 8^th^ band in the multiplex PCR span a *MseI*‐restriction site. The threshold for WGA products to proceed to downstream analysis was set at QC‐5 and higher.

To evaluate storage possibilities to enable postponed processing, single cells were frozen in PBS or in *Ampli*1™ lysis buffer (ALB). High‐quality *Ampli*1™ WGA product, with maximal QC scores, could still be obtained when cells were frozen down directly after lysis and subsequently thawed after 72 h (success rate (SR): PBS *n* = 1/3, ALB *n* = 2/3), 1 week (SR: PBS *n* = 2/3, ALB *n* = 2/3) and after 1 month (SR: PBS *n* = 2/3, ALB *n* = 0/3).

**Fig. 2:**
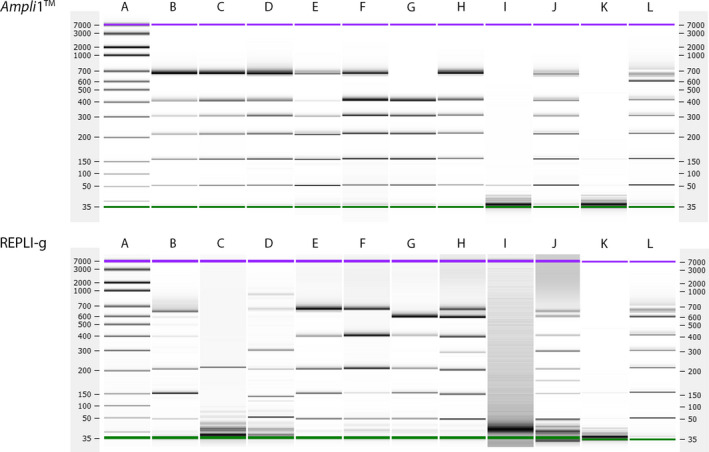
VyCAP QC scores of seven MCF‐7 samples ranging from 1 to 10 cells, after *Ampli*1^TM^ WGA (B‐H, top panel) and REPLI‐g WGA (B‐H, bottom panel). Generated QC multiplex PCR products were visualized on the Bioanalyzer platform using DNA High Sensitivity chips. (A) DNA HiSense ladder, (B‐D) single MCF‐7 cells, (E‐F) pool of two MCF‐7 cells, (G) pool of 5 MCF‐7 cells (H) pool of 10 MCF‐7 cells, (I) WGA negative control, (J) WGA positive control containing 1 ng genomic DNA, (K) negative control, (L) positive control.

MCF‐7 cells amplified with the *Ampli*1™ WGA Kit mostly attained the maximum quality control score of QC‐7, except for the 5‐cell pool which had QC‐5 (Fig. 2, top panel). However, much lower QC scores were observed for REPLI‐g WGA products (Fig. 2, bottom panel). In fact, WGA products of single cells amplified with REPLI‐g rarely produce QC‐5 or higher. Although the quality of the REPLI‐g WGA products seems to increase with the number of cells punched, only the REPLI‐g WGA product of 10 cells obtained the maximum attainable QC‐7. Contrarily, *Ampli*1™ WGA products from single MCF‐7 cells reached a maximum QC‐7 with a 72% success rate (37 out of 52 reactions; required to obtain all samples required for sequencing of sufficient quality). Our results show that the *Ampli*1™ WGA method is superior to REPLI‐g WGA for the amplification of CellSearch‐enriched, VyCAP‐isolated single cells.

### 3.2 *Ampli*1™ LowPass versus TruSeq PCR‐free library preparation on *Ampli*1^TM^ WGA products

After determining that the *Ampli*1™ WGA method is the superior WGA method for CellSearch‐enriched, VyCAP‐punched single cells, this method was used to perform WGA on two punching series: 4 × 1 leukocyte, 2 × 2 leukocytes, 2 × 5 leukocytes and 2 × 10 leukocytes (Table 1, samples 1‐10), and 3 × 1 MCF‐7 cell, 2 × 2 MCF‐7 cells, 1 × 5 MCF‐7 cells and 1 × 10 MCF‐7 cells (Table 1, samples 11‐17). In addition to these samples, we included samples with suboptimal QC scores (Table 1, samples 18‐20), WGA samples with a maximum QC score supplied by VyCAP (Table 1, samples 21 and 22), genomic MCF‐7 DNA amplified with *Ampli*1™ (Table 1, sample 23) and nonamplified MCF‐7 DNA (Table 1, sample 24). Libraries of all 24 DNA samples were prepared with the TruSeq PCR‐free method. Only 23 sequencing libraries were prepared using the *Ampli*1™ LowPass method because nonamplified MCF‐7 DNA does not have the *Ampli*1™ adaptors required for this method. After library preparation, library fragment sizes were measured. Samples with quantifiable amounts of DNA and with a modal size around 700 bp were considered successfully prepared NGS libraries. During library preparation with the *Ampli*1™ LowPass Kit, 8 out of 24 samples failed and required one to three additional attempts. All 24 libraries prepared with the TruSeq PCR‐free kit were successful at first try. Although the concentrations of the libraries were sufficient after preparation with both methods, the yield of the *Ampli*1™ LowPass Kit was much higher (15.3–80.7 nm; IQR 45.4 nm; Table 1) than the yield of the TruSeq PCR‐free kit (3.8–8.8 nm; IQR 5.9 nm; Table 1; paired Wilcoxon, *P* = 7.15*10^−7^).

**Fig. 3:**
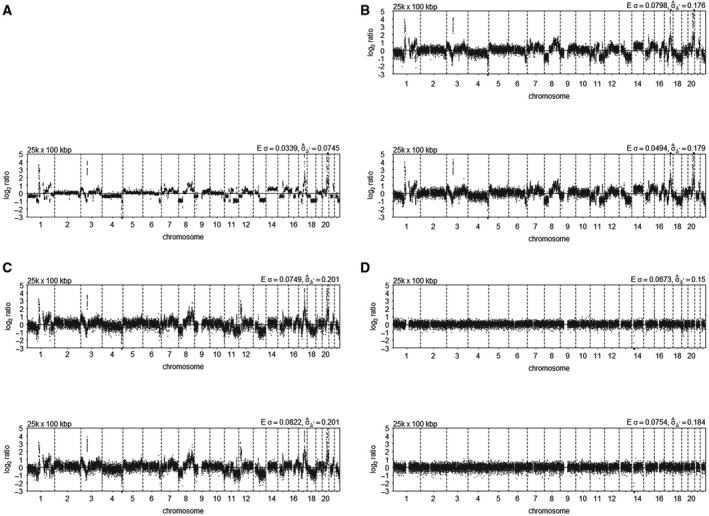
CN profiles of *Ampli*1 WGA samples after TruSeq PCR‐free or *Ampli*1 LowPass library preparation compared with MCF‐7 DNA without amplification. The CN profiles were plotted with QDNAseq on default settings. (A) Genomic MCF‐7 DNA (Table 1, sample 24), (B) *Ampli*1 WGA of MCF‐7 DNA (Table 1, sample 23), (C) a single MCF‐7 cell (representative of *n* = 3, Table 1, sample 13) and (D) a single HBD leukocyte (representative of *n* = 4, Table 1, sample 2). Top of each panel: *Ampli*1 LowPass libraries, bottom of each panel: TruSeq PCR‐free libraries.

After sequencing, the noise in the sequenced data was determined by calculating the median absolute deviation (MAD) of normalized reads from chromosome 2 for MCF‐7 cells and genome wide for leukocytes. For MCF‐7, only chromosome 2 was used since this chromosome is minimally affected by ploidy changes [28]. Lower MADs were obtained by the *Ampli1* ^TM^ LowPass Kit in 12 out of 23 reactions, although the differences are not significant (paired Wilcoxon, *P* = 0.79 for MCF‐7 and *P* = 0.15 for leukocytes; Tables 3 and 4). Thus, MADs obtained with the two library preparation methods were similar and representative results are depicted in Fig. 3. Importantly, samples with QC‐5 or QC‐6 had MADs comparable to samples with QC‐7 (Tables 3 and 4, sample no. 18−20 and Fig. S2), demonstrating that samples with QC‐5 are of sufficient quality to sequence and thus served as a minimal threshold to sequence subsequent WGA samples.

**Table 3:** Comparison of *Ampli*1 LowPass and TruSeq PCR‐free library preparation methods by MAD measured genome wide in leukocytes of HBD0. Samples with lower MADs have green fills. *Wilcoxon signed rank *P* = 0.15. No, number; MAD, mean absolute deviation.

Sample No.	Name	MAD TruSeq PCR‐free*	MAD *Ampli*1 LowPass*
1	Leuko, 1A	0.198	0.169
2	Leuko, 1B	0.194	0.162
3	Leuko, 1C	0.220	0.214
4	Leuko, 1D	0.277	0.244
5	Leuko, 2A	0.237	0.235
6	Leuko, 2B	0.255	0.236
7	Leuko, 5A	0.236	0.226
8	Leuko, 5B	0.201	0.217
9	Leuko, 10A	0.295	0.296
10	Leuko, 10B	0.216	0.226
18	Leuko, QC‐6	0.219	0.223

**Table 4:** Comparison of *Ampli*1 LowPass and TruSeq PCR‐free library preparation methods by MAD measured on chromosome 2 of MCF‐7 cells. Samples with lower MADs have green fills. *Wilcoxon signed rank *P* = 0.79. No., number; MAD, mean absolute deviation.

Sample No.	Name	MAD TruSeq PCR‐free*	MAD *Ampli*1 LowPass*
11	MCF‐7, 1A	0.292	0.283
12	MCF‐7, 1B	0.292	0.266
13	MCF‐7, 1C	0.240	0.245
14	MCF‐7, 2A	0.260	0.266
15	MCF‐7, 2B	0.260	0.268
16	MCF‐7, 5	0.266	0.275
17	MCF‐7, 10	0.241	0.230
19	MCF‐7, QC‐6	0.237	0.248
20	MCF‐7, QC‐5	0.309	0.204
21	VyCAP Ctrl 1	0.260	0.320
22	VyCAP Ctrl 2	0.448	0.431
23	MCF‐7, Ampli1	0.182	0.186

Despite the low quality of the REPLI‐g WGA products, we have verified their CNV profiles after library preparation using the TruSeq Kit for a few samples. As expected, the libraries produced poor quality CNV profiles as demonstrated by higher MADs for all samples (paired Wilcoxon, *P* = 0.03; Table 5). The variance decreased with higher number of cells in the sample (Fig. S3, σ̂_Δ_), similar to the QC scores of the REPLI‐g WGA products. These results show that REPLI‐g on fixed single cells produces CNV profiles of poor quality, and therefore, this method is not suited for WGA of patient‐derived CTCs isolated through the CellSearch enrichment and VyCAP punching.

**Table 5:** Comparison of *Ampli*1 and REPLI‐g WGA methods by MAD measured on chromosome 2 of MCF‐7 cells. Samples with lower MADs have green fills. *Wilcoxon signed rank *P* = 0.03. No, number; MAD, mean absolute deviation; n.a. not available.

Sample No.	Name	MAD *Ampli*1 + TruSeq*	MAD REPLI‐g + TruSeq*
11	MCF‐7, 1A	0.292	0.769
12	MCF‐7, 1B	0.292	0.534
13	MCF‐7, 1C	0.240	Failed sequencing
14	MCF‐7, 2A	0.260	0.763
15	MCF‐7, 2B	0.260	n.a.
16	MCF‐7, 5	0.266	1.114
17	MCF‐7, 10	0.241	0.286
24	MCF‐7, Genomic	0.062	0.107

After *Ampli*1^TM^ amplification, the normalized read counts in 100 kb bins of 1 ng of MCF‐7 DNA strongly correlate with nonamplified genomic MCF‐7 DNA (Spearman *r* = 0.937 with TruSeq‐prepared libraries, *r* = 0.911 with LowPass‐prepared libraries), which demonstrates uniform amplification by the *Ampli*1™ method. TruSeq versus *Ampli*1™ LowPass library preparations from the same sample also correlated strongly regardless of input material (Spearman *r* = 0.929 with 1 ng MCF‐7 DNA, *r* = 0.927, *r* = 0.991 and *r* = 0.981 for each of the single MCF‐7 cells, samples 11, 12 and 13). Furthermore, amplification of single MFC‐7 cells with *Ampli*1™ also correlates with nonamplified genomic MCF‐7 DNA regardless of the chosen library preparation method (Spearman *r* = 0.777, *r* = 0.767 and *r* = 0.845 for single‐cell libraries prepared with *Ampli*1™ LowPass, *r* = 0.784, *r* = 0.797 and *r* = 0.859 for single‐cell libraries prepared with TruSeq PCR‐free); however, the correlation across single cells irrespectively of library preparation method is consistently lower. This lower correlation is likely caused by subclonality of the MCF‐7 cell line in combination with amplification bias due to the low amount of DNA present in single cells. Our results show that library preparation from *Ampli*1™ WGA products made compatible with the TruSeq PCR‐free method had comparable MADs and a higher success rate than *Ampli*1™ LowPass‐prepared libraries. Therefore, the *Ampli1*™ WGA method followed by the Illumina Truseq PCR‐free Library Preparation Kit was selected as the preferred pipeline to generate CNV profiles from patient‐derived single CTCs.

### 3.3 Validation of the single CTC CNV profiling pipeline

Four patients (i.e. two males with prostate cancer (PC1 and PC2) and two females with breast cancer (BC1 and BC2)) were selected to validate our single CTC CN profiling pipeline. Peripheral blood of these patients was processed following the CellSearch method and resulted in the following CTC counts per 7.5 mL of blood: 705 for PC1, 108 for PC2, 197 for BC1 and 6422 for BC2. For the breast cancer patients, the enriched CTC fraction yielded by the CellSearch CX‐9 protocol was transferred to a VyCAP microwell chip. For the prostate cancer patients, the cell fraction present in the MagNest Cartridge was collected and transferred to a VyCAP chip. The number of CK+, DAPI+, CD45‐ events present on the microchip was *n* = 163 for PC1 (recovery rate, RR = 23% compared to CellSearch), *n* = 54 for PC2 (RR = 50%), *n* = 159 for BC1 (RR = 81%) and *n* = 3560 for BC2 (RR = 55%). For 87 out of a total of 214 WGA reactions (41%), the required QC ≥ 5 was reached. For each patient, we selected the WGA products of 10 single CTCs with the highest QC scores for subsequent library preparation and sequencing (Table 6). For the single CTCs of PC1, only one cell reached the maximum quality control score of QC‐7, two cells reached QC‐4, and the remaining seven cells reached QC‐3. Despite the low‐quality WGA products in this case, these samples were not excluded from the analysis to further evaluate the effect of a low QC score on the downstream results in these patient‐derived samples. The three other patients had WGA products of 10 single cells (BC1), 8 single cells (BC2) and 8 single cells (PC2) with ≥ QC‐5 out of the 10 for further sequencing. All subsequent TruSeq PCR‐free library preparations of these patient‐derived single cells were successful (i.e. modal size of 700 bp according to the Bioanalyzer and more than 2 nm library yield).

**Table 6:** Overview of patient‐derived CTCs for validation of the pipeline. Samples are grouped per patient. VyCAP QC: scores can range from QC‐0 to QC‐7. Ampli1 QC: scores can range from QC‐0 to QC‐4. No, number.

Sample No.	Sample Name	Sample Type	Donor/Source	Gender	Input	VyCAP QC score	Ampli1 QC score	Yield TruSeq (nm)
49	BC1‐1	CTC	BC1	Female	1 cell	7	4	2.21
50	BC1‐2	CTC	BC1	Female	1 cell	7	4	3.85
51	BC1‐3	CTC	BC1	Female	1 cell	7	4	3.32
52	BC1‐4	CTC	BC1	Female	1 cell	7	4	2.95
53	BC1‐5	CTC	BC1	Female	1 cell	7	4	3.34
54	BC1‐6	CTC	BC1	Female	1 cell	7	4	4
55	BC1‐7	CTC	BC1	Female	1 cell	7	4	4.26
56	BC1‐8	CTC	BC1	Female	1 cell	7	2	5.87
57	BC1‐9	CTC	BC1	Female	1 cell	7	1	5.91
58	BC1‐10	CTC	BC1	Female	1 cell	7	1	3.79
59	BC2‐1	CTC	BC2	Female	1 cell	7	3	4.34
60	BC2‐2	CTC	BC2	Female	1 cell	7	2	4.54
61	BC2‐3	CTC	BC2	Female	1 cell	5	2	10.42
62	BC2‐4	CTC	BC2	Female	1 cell	6	2	11.89
63	BC2‐5	CTC	BC2	Female	1 cell	7	3	6.3
64	BC2‐6	CTC	BC2	Female	1 cell	5	1	6.03
65	BC2‐7	CTC	BC2	Female	1 cell	7	3	6.33
66	BC2‐8	CTC	BC2	Female	1 cell	6	3	5.82
67	BC2‐9	CTC	BC2	Female	1 cell	5	1	4.23
68	BC2‐10	CTC	BC2	Female	1 cell	7	1	6.8
69	PC1‐1	CTC	PC1	Male	1 cell	3	1	8.98
70	PC1‐2	CTC	PC1	Male	1 cell	3	1	15.06
71	PC1‐3	CTC	PC1	Male	1 cell	4	1	8.29
72	PC1‐4	CTC	PC1	Male	1 cell	3	1	13.66
73	PC1‐5	CTC	PC1	Male	1 cell	7	3	8.77
74	PC1‐6	CTC	PC1	Male	1 cell	3	2	8.88
75	PC1‐7	CTC	PC1	Male	1 cell	3	1	7.68
76	PC1‐8	CTC	PC1	Male	1 cell	4	2	7.84
77	PC1‐9	CTC	PC1	Male	1 cell	3	0	18.18
78	PC1‐10	CTC	PC1	Male	1 cell	4	1	3.96
79	PC2‐1	CTC	PC2	Male	1 cell	5	2	8.53
80	PC2‐2	CTC	PC2	Male	1 cell	7	4	12
81	PC2‐3	CTC	PC2	Male	1 cell	7	4	4.84
82	PC2‐4	CTC	PC2	Male	1 cell	3	1	8.8
83	PC2‐5	CTC	PC2	Male	1 cell	7	4	6.54
84	PC2‐6	CTC	PC2	Male	1 cell	7	4	5.84
85	PC2‐7	CTC	PC2	Male	1 cell	7	4	9.04
86	PC2‐8	CTC	PC2	Male	1 cell	4	2	10.65
87	PC2‐9	CTC	PC2	Male	1 cell	7	4	6.65
88	PC2‐10	CTC	PC2	Male	1 cell	4	0	5.97

### 3.4 CNV calling and minimal sequencing depth

After sequencing, four patient‐derived single cells were rejected by QDNAseq, despite passing NEBNext and Bioanalyzer library prep QC. The data from these cells (i.e. BC1‐9, BC1‐10, PC1‐2 and PC1‐9) were of insufficient quality and could not be normalized by the algorithm. Remarkably, two out of the four rejected samples had a QC‐7. Besides these four samples, samples BC2‐3, PC1‐5, PC1‐10 and PC2‐4 deviate from the rest of the samples regarding the number of ‘empty’ bins (Fig. S4) and were excluded from further analysis. Sequencing data from the remaining 32 single cells were included in subsequent heterogeneity analysis.

**Fig. 4:**
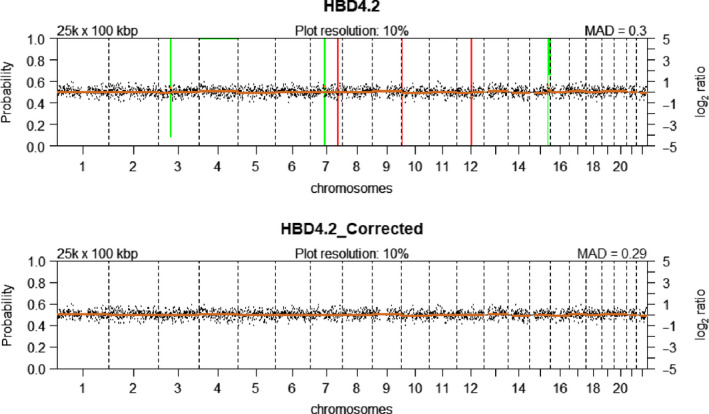
CNV plots of a HBD leukocyte (representative of *n* = 13, Table 2, sample 38) before and after wave bias correction using NoWaves. (Top panel) uncorrected CNV profile showing six CN alterations. (Bottom panel) corrected CNV profile showing no CN alterations. Green bars represent segments with CN gains, and red bars represent segments with CN loss. The probability of the gain/loss is represented by the length of the bars.

Sequencing data may contain technical artefacts appearing as waves in plotted CN profiles. Although the exact cause remains to be elucidated, this ‘wave bias’ is correlated with GC‐content [37]. To counter these artefacts, the NoWaves software package [32] was used to generate our own calibration set of 24 leukocyte profiles derived from six HBDs (3 males: HBD2, HBD4 and HBD5; 3 females: HBD1, HBD3 and HBD6; four single leukocytes per HBD; Table 2). First, the CNV profile of every single leukocyte was regressed against the remaining 23 leukocyte profiles. Subsequently, the leukocytes with a variance above 0.05 (*n* = 11), as determined by the NoisePlot function, were excluded from the calibration set. The final calibration set consisted of 13 leukocyte profiles from six HBDs. Prior to correction by NoWaves, a total of 98 segments (i.e. consecutive nondeviating bins on the same chromosome are merged into one segment) were observed cumulatively in the 13 leukocytes. Applying the NoWaves correction resulted in an almost twofold reduction of the number of segments to 55 (representative results in Fig. 4), demonstrating effective removal of wave bias in sequence data of single cells, which ultimately allows more accurate detection of true breakpoints.

Stringent thresholds suited for CNV analysis of single cells (due to the integral presence of chromosomes) were set in CGHcall, after which CNV regions in the NoWaves‐corrected profiles were called. The minimum sequencing depth needed to reliably generate CN profiles from single cells at a 100‐kb resolution was determined on three samples: one single MCF‐7 cell (sample 13), *Ampli*1‐amplified MCF‐7 DNA (sample 23) and BC1‐4 (sample 52). The number of reads from these three samples was subsampled to 0.5 m, 1 m, 2.5 m and 5 m for ten iterations. All subsampling iterations resulted in near‐identical CNV profiles (Fig. S5) with high correlations to the original samples at 5 m and 2.5 m reads for all samples: mean Spearman *r* = 0.965 and *r* = 0.934 for the single MCF‐7 cell, mean Spearman *r* = 0.952 and *r* = 0.897 for *Ampli*1‐amplified MCF‐7 DNA, and mean Spearman *r* = 0.999 and *r* = 0.992 for BC1‐4 (Table S2). At 1 m reads, CNV profiles were mostly similar and most characteristic aberrations could be detected. Furthermore, at 1 m reads, chromosome arm copy number was occasionally misclassified and focal aberrations were missed when compared to the original sample (mean Spearman *r* < 0.9 for all iterations). At 0.5 m reads, the CNV profiles of the ten subsamples produced varying CNV profiles per sample. Altogether, a minimum of 2.5 m mapped paired‐end reads of 100 bp is recommended to accurately produce CNV profiles of single cells, whereas 1 m or less reads are insufficient.

To compare the results of our pipeline, we analysed our data using the open‐source web platform ‘Ginkgo’ [21], which is developed specifically to analyse CNVs from single cells. This platform requires bed files to produce CNV profiles; however, uploading of files larger than 1 GB, equivalent to bed files larger than 10 m reads, is restricted. For our sequencing data, Ginkgo consistently overrated the ploidy of the samples with higher read counts, demonstrating suboptimal normalization. Furthermore, contrarily to our pipeline, Ginkgo gives an absolute CN call based on read counts relative on the rest of the pool, which misclassifies samples with more sequenced reads as having genome‐wide gains. In our pipeline, each sample is normalized and subsequently corrected using a calibration set, which reduces false‐positive calls. Calls are limited to relative gains and losses to better represent true CN alterations.

### 3.5 Retrospective detection of low‐quality samples after WGA

The sequencing data from four patient‐derived single cells (*i.e*. BC1‐9, BC1‐10, PC1‐2 and PC1‐9) could not be normalized by the QDNAseq algorithm. This was despite a QC‐7 for BC1‐9 and BC1‐10 and despite comparable library fragment sizes and yield compared with successfully sequenced samples. Additional assessment of WGA product quality using the *Ampli1™* QC Kit shows that *Ampli1™* QC is more accurate at identifying poor quality WGA samples compared with VyCAP QC. However, *Ampli1™* QC underestimates the QC score of high‐quality samples (Table 6). To more conclusively assess the *Ampli1*™ WGA product quality, we have employed the mFAST‐SeqS method on 1 ng of WGA product in a subset of 24 samples and 12 HBD leukocytes to evaluate the distribution of LINE‐1‐derived reads per chromosome. For BC1‐9, BC1‐10, PC1‐2 and PC1‐9, the sequence reads were disproportionately distributed as compared with the patient‐derived CTCs that generated high‐quality CN profiles as well as the leukocytes (Figs. S6,S7). For these four samples, nearly all sequence reads were mapped to only a few chromosome arms, whereas other chromosome arms had nearly no mapped reads (Table S3). In contrast to the VyCAP QC and the *Ampli1*™ QC, only mFAST‐SeqS was able to correctly predict for all 36 (100%) WGA products whether successful CN profiles could ultimately be generated. This prediction accuracy was only 29/36 (81%) for VyCAP QC and 26/36 (72%) for *Ampli1*™ QC.

### 3.6 Assessment of intrapatient heterogeneity

The primary objective of this study was to establish a method to enable the assessment of intrapatient CNV heterogeneity at single CTC level. As mentioned, CTCs of two PC patients and two BC patients were collected. Unfortunately, we had to exclude one patient (PC1) from the analysis because for this patient four samples failed to meet the determined quality thresholds (PC1‐1, PC1‐4, PC1‐6 and PC1‐7), the sequencing libraries of two samples were of insufficient quality (PC1‐5 and PC1‐10), and two samples could not be normalized (PC1‐2 and PC1‐9). The two remaining CTCs from PC1 had sufficient overall quality as determined by low MAD and a comparable number of empty bins to successful samples, but would not contribute in our effort to assess intrapatient heterogeneity.

**Fig. 5:**
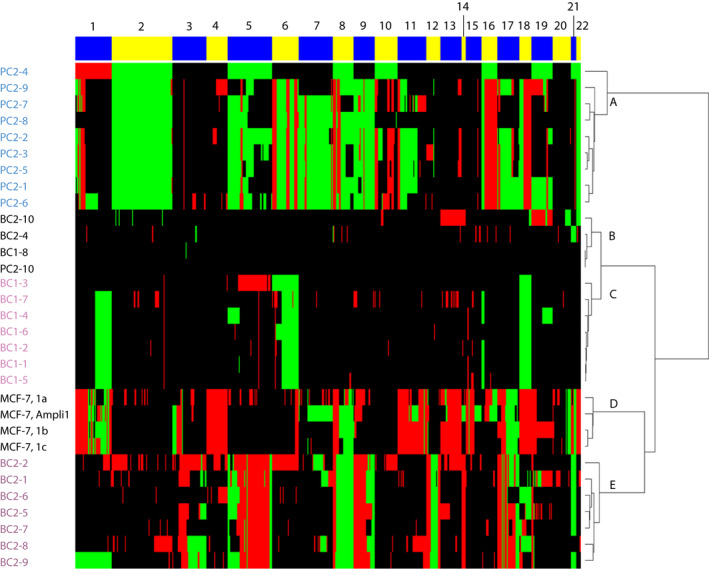
Heatmap of all successfully generated CN profiles from patient‐derived single CTCs (BC1, 8 samples; BC2, 9 samples; PC2, 10 samples) and MCF‐7 (3 single‐cell samples and 1 genomic DNA sample) after *Ampli*1 WGA. Hierarchical clustering of CN profiles called by CGHcall resulted in (A) a PC2 patient‐specific cluster, (B) a cluster with no/low CN alterations, (C) a BC1 patient‐specific cluster, (D) an MCF‐7 cluster and (E) a BC2 patient‐specific cluster. Red bars represent CN losses, and green bars represent CN gains. The numbers above the alternating yellow and blue bars indicate chromosome numbers.

To investigate the similarity between the patient‐derived single‐cell CNV profiles, unsupervised weighted clustering of called CNV data on eight cells of BC1, nine cells of BC2, nine cells from PC2 and 3 single MCF‐7 cells together with *Ampli*1™‐amplified genomic DNA of MCF‐7 was performed. Hierarchical clustering of the CNV profiles resulted in two large clusters: one containing only PC2‐derived CTCs (Fig. 5, cluster A) and a second containing all other samples. This second cluster can be broken down further into two groups: one with few CNVs and one with many CNVs. The ‘few‐CNV’ group can be subdivided into a group of patient‐derived cells harbouring very few CN alterations and originating from each of the three patients (Fig. 5, cluster B) and a cluster containing only CTCs from the ER+/HER2‐ BC1 (Fig. 5, cluster C). The ‘many‐CNV’ group can be separated into MCF‐7‐derived samples (Fig. 5, cluster D) and CTCs from ER‐/HER2+ BC2 (Fig. 5, cluster E).

Consecutive regions with an equal CNV‐call in all samples are plotted as a single condensed segment in our clustering method. Therefore, the sizes of the chromosomes in Fig. 5 are not plotted to true scale. Instead, the size of the chromosomes represents the number of informative segments of the included samples. When looking at the patient‐derived CNV profiles, heterogenic regions can be observed. Notably CTCs of BC2 show CNV heterogeneity at a high frequency, mainly in chromosome 17, which harbours the *BCRA1*, *TP53, ERBB2* and *NCOR1* genes. BC1 has a subclonal CTC (BC1‐4) with gains at chromosomes 5 and 19 that were not found in the other CTCs from this patient. PC2 CTCs were divided into four different clusters, presumably due to heterogeneity for chromosomes 8 and 17q that harbour the *MYC* and *NGFR* genes. Furthermore, the CNV profiles of the three MCF‐7 cells differ from the profile derived from genomic MCF‐7 DNA. These findings are consistent with the previously described genetic variation in the karyotype of MCF‐7 cell lines [38]. One cluster contains profiles with very few CN alterations that were derived from each of the three patients. These cells were classified as CTCs based on CellSearch characteristics, but show no genomic alterations. Likely, these cells are nontumour circulating epithelial cells that enter the blood along with CTCs [39]. Furthermore, a few samples (i.e. Fig. 5, samples BC2‐4, BC1‐7 and MCF‐7‐1a) display a multitude of small losses even after corrections by our bioinformatics pipeline. These losses are presumably sample‐specific false‐positive losses caused by regions with low coverage. Despite these false‐positive losses, the clustering of CTCs per patient and the successful assessment of variations in CN alterations within one patient demonstrate the feasibility to assess intrapatient heterogeneity with our single‐cell CNV pipeline.

### 3.7 CNVs in clinically relevant regions

**Fig. 6:**
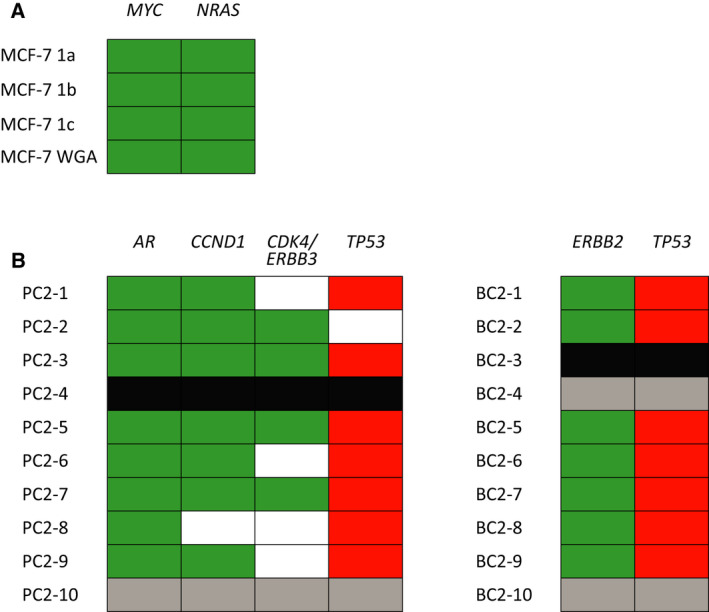
Selection of clinically relevant amplifications identified among patient‐derived single CTCs from 2 cancer patients (BC2, 9 samples; PC2, 9 samples) and MCF‐7 (3 single‐cell samples, 1 genomic DNA sample) after *Ampli*1 WGA. (A) Amplification of *MYC* (70 bins) and *NRAS* (13 bins) in three single MCF‐7 cells and *Ampli*1‐amplified MCF‐7 DNA. (B) Amplification of *AR* (14 bins), *CCND1* (16 bins), *CDK4/ERBB3* (23 bins) and *ERBB2* (3 bins). Bin sizes were set to 100 kb for all samples. Green fills indicate amplifications, red fills indicate losses, black fills indicate excluded samples, grey fills indicate samples that clustered as ‘leukocyte‐like’, and white fills represent CN‐neutral regions. PC2‐4 had insufficient resolution for these loci and was excluded.

In addition to the assessment of genome‐wide CNVs per patient, heterogeneity in clinically relevant regions was evaluated in more detail (detailed results in Fig. S8). First, the CNV profile of MCF‐7 was analysed. Clear amplifications of the *MYC* gene (70 100 kb bins) and *NRAS* gene (13 100 kb bins) were called, in concordance with previously described delimited amplifications of these genes [35] (Fig. S8A). Next, we looked for clinically actionable gene amplifications in the three patients and the MCF‐7 samples that have been previously described by the OncoKB database [40]. Amplifications of *CDK4/ERBB3*, *CCND1* and *ERBB2* as well as loss of *TP53* could be detected in PC2 and BC2 (Fig. 6B). In our cluster analysis, the X and Y chromosomes were excluded to allow for the comparison of samples regardless of gender. We also assessed the heterogeneity of *AR* CNVs by rerunning our bioinformatic pipeline using only leukocytes derived from males HBDs (*n* = 9). PC2 showed high variation in *AR* amplification in CTCs of this single patient (log2 range 1.47‐5.72; Fig. S8E), which demonstrates the feasibility of our pipeline to determine CN aberrations on the X chromosome. Importantly, our pipeline can detect clinically actionable amplifications of *ERBB2*, *ERBB3*, *CDK4*, *CCND1*, *NRAS*, *MYC* and *AR* in single cells. Moreover, our pipeline can be employed to detect other (clinically relevant) CN aberrations such as *MDM2* gain, *FGFR1* gain, *TP53* loss and *SMARCB1* loss down to 300 kb.

## 4 Discussion

### 4.1 Technical and bioinformatic design

Here, we established and validated an end‐to‐end pipeline to obtain CN profiles from patient‐derived single CTCs which enables the assessment of intrapatient heterogeneity. When designing this pipeline, technical notabilities were encountered.

First, it was shown that *Ampli*1^TM^ WGA was superior to REPLI‐g WGA for mildly fixed single cells using the CellSearch protocol, which confirms previous results by Babayan et al. [41]. Our results suggest that the DNA‐crosslinking fixative present in CellSave tubes is too rigorous and blocks phi29 polymerase used in the REPLI‐g WGA, which makes REPLI‐g WGA unsuitable for the amplification of CellSave‐fixed CTCs. The *Ampli*1™ Kit circumvents DNA crosslinks by enzymatic digestion of the genome prior to adaptor‐based amplification. Because *Ampli*1™ WGA is limited to DNA fragments digested by *MseI*, chromosomal regions which are CG‐rich or CG‐poor can have poorer coverage. However, with a coverage of 74%, *Ampli*1™ WGA has a higher coverage than other single‐cell WGA methods [42, 43].

Although not all DNA fragments after *MseI* digestion will be amplified successfully, *Ampli*1™ WGA followed by low‐pass sequencing suffices for copy number profiling with 100 kb bin sizes. Using MCF‐7, we showed uniform amplification of *Ampli*1™ WGA and a median Spearman correlation between single cells after WGA and genomic MCF‐7 DNA of Spearman *r = *0.812 (Table S4), whereas 1 ng of MCF‐7 DNA has a Spearman correlation with genomic MCF‐7 DNA of *r* = 0.937. A main contributor of this lower correlation is likely subclonality of the MCF‐7 cell line. The median correlation of *r = *0.812 is comparable with the median correlation of *r = *0.809 between 27 MCF‐7 cell line strains collected from different laboratories around the world by Ben‐David et al. [38] (Table S5). However, we cannot formally exclude the possibility of amplification bias during WGA due to small amounts of DNA when amplifying single cells.

Second, library preparations with the TruSeq PCR‐free method were more frequently successful than Ampli1 LowPass library preparations. The recommended input for the TruSeq method was only obtained after reamplification of our WGA products, since this method requires more DNA to allow PCR‐free attachment of adapter fragment in favour of ligation. Using the TruSeq method all prepared libraries passed QC (i.e. library size and yield) on the first attempt. The Ampli1 LowPass library preparation, however, is dependent on multiple clean‐up steps followed by adaptor attachment steps by polymerase extension prior to amplification. This method can generate much higher yields than the TruSeq method when the two initial clean‐up steps and adapter attachments are successful. With varying input amounts and a high dependency of DNA binding during the initial two adapter attachments prior to amplification, library preparations with Ampli1 LowPass can also fail if DNA binding falls short. Unfortunately, successful clean‐up and adapter attachment can only be verified at the end of the entire procedure. In our hands, 8 out of 24 samples failed (i.e. no yield) and required one to three additional attempts using the LowPass method.

Third, when validating the established pipeline on patient CTCs, the sequencing data from four single CTCs could not be normalized and had to be excluded, even though two of these samples had a QC‐7. This is presumably due to inefficient *MseI* digestion. Partially digested DNA from single cells results in longer fragments that are not amplified (efficiently) resulting in unevenly distributed amplification, yielding low‐complexity libraries that pass QC. We employed the mFAST‐SeqS method as an additional QC to assess uniform amplification of chromosome arms in the WGA product. We observed a nonuniform distribution of our sequence reads in the four samples that failed sequencing: only a few chromosome arms contained almost all reads. These findings explain high‐quality control scores and yield from poorly amplified samples. This finding also supports our hypothesis of partial digestion by the *MseI* enzyme resulting in poor sequencing libraries, undetected by multiplex‐based quality controls. Therefore, to obtain sequencing libraries of sufficient quality, the digestion needs to be done for 3 h according to the manufacturer of *MseI*, rather the 5 min as described by the *Ampli*1™ protocol [44].

### 4.2 Comparison of results to other single CTC analysis methods

The VyCAP punching system was previously described by Andree et al. to isolate viable single patient‐derived CTCs. Unfortunately, the use of unfixated cells as input material resulted in a substantial loss of detectable CTCs compared with the parallel enumeration through the CellSearch method [45]. Albeit with a lower success rate, their results demonstrate it is possible to isolate viable CTCs using the VyCAP puncher, which can subsequently be analysed with our pipeline. Success rates of our pipeline vary per patient and are possibly coupled to CTC vitality. Our success rate of 41% is lower when compared to other studies which also used LA‐PCR on CellSearch‐enriched samples. Polzer et al. and Neves et al. obtained a 68% and 65.1% success rate with at least QC score three out of four of the Ampli1 QC Kit in breast cancer patients [46, 47]. Unfortunately, we were unable to obtain 10 single CTCs of sufficient quality from one of our included prostate cancer patients. From the remaining patients, however, we obtained 26 copy number profiles out of 30 successful WGA reactions (85%). With mFAST‐SeqS, we would have been able to identify these samples prior to sequencing, demonstrating the benefits of a mFAST‐SeqS‐based quality control. When looking at one of the key papers written on this subject by Chemi et al. [39], the success rate of whole genome sequencing of single CTCs of one patient was 21%, which is little more than half of our success rate of 41%. Strikingly, this paper also identified CTCs based on CellSearch characteristics that did not harbour genomic alterations when looking at the shallow whole genome sequencing data of these ‘false‐positive’ CTCs. They report these cells as circulating epithelial cells (CECs) and suggest that the false‐positive enumeration of these cells as CTCs can lead to bias and a underestimation of the predictive value of the presence of ‘real’ CTCs. Chemi et al. therefore conclude that together, CTC enumeration and genomic profiling highlight the potential of CTCs as early predictors of NSCLC recurrence.

Our finding of CellSearch‐positive cells which turn out to have no CNVs supports the findings of Chemi et al. and again highlights the necessity of genomic analysis on CellSearch‐enriched CTCs to gather reliable information on both CTC count and molecular characteristics of the (minimal residual) disease in a patient. In this respect, the mFAST‐SeqS QC performed on WGA products allows verification of uniform amplification as well as the detection of samples with normal karyotypes and samples with CNVs for the detection of CTCs. This way mFAST‐SeqS can help to avoid the futile analysis of samples without CNVs, which Ampli1 and modified VyCAP QC cannot. Therefore, to obtain the highest success rate after sequencing, we recommend the mFAST‐SeqS QC instead of the other two QCs, despite its higher cost. However, in practice the decision which QCs are indicated might be more pragmatic and should depend on the consideration of time, costs and the number of CTCs available.

To accurately produce CNV profiles of single cells, a minimum of 2.5 m mapped paired‐end reads of 100 bp are recommended for our pipeline. However, it was previously shown that as few as 200 000 reads at a bin size of 100 kb was sufficient to detect CN aberrations and this was comparable with 3.5 m reads at this bin size [44]. We randomly subsampled our data for ten iterations per sample at 0.5 m reads, 1 m reads, 2.5 m reads and 5 m reads. Random subsampling of our data from approximately 15 m reads to 2.5 m reads leads to accurate CN profiles with our pipeline, with highly correlated profiles at each iteration. We were unable to obtain reproducible CN profiles with 1 m reads or less at a bin size of 100 kb. A lower number of reads at this bin size introduces false‐positive and false‐negative CNVs, and this should be avoided in the detection of heterogeneity in single CTCs [44].

### 4.3 The advantages and challenges of copy number profiling of single CTCs

The major advantage of our pipeline is that it is constructed of five individually adjustable steps, namely CTC enrichment, CTC isolation, whole genome amplification, low‐pass sequencing and bioinformatic analysis. This design ensures technical as well as practical flexibility as it can be adjusted according to specific research needs and it can be conveniently carried out in multiple facilities to ease implementation into the research setting and, perhaps eventually, clinical practice. For the first step, we chose to apply the CellSearch method using paramagnetic α‐EpCAM antibodies as this is the current gold standard for CTC enrichment. However, CellSearch enrichment is also possible with other antibodies (e.g. α‐MCAM [48]), depending on the research question. Regarding the chosen single‐cell isolation method, other frequently used techniques include FACS sorting and the DEParray method [12, 49]. However, FACS sorting requires an input with a relatively high number of cells of interest to enable reliable sorting and while DEParray does enable the isolation of rare cells, it is restricted to a collection volume of 13 µL. In contrast, the VyCAP puncher system can isolate and collect rare cells into a 1 µL volume. An additional benefit of the VyCAP isolation method is the possibility to use different types of input material. As our main aim was to validate our pipeline on patient‐derived CTCs, we chose to include patients previously known to have high CTC count. To increase the method’s sensitivity, an interesting possibility is the use of diagnostic leukapheresis material as input material. Leukapheresis is a previously described method to increase the CTC detection rate when patients CTC counts in the regular CellSearch measurement in 7.5mL of blood are low [50]. After the input material has been chosen, enrichment of samples can be performed through any type of fluorescent staining or through the use of cell size‐based techniques (e.g. Parsortix or RosetteSep [17, 51, 52]). Therefore, the VyCAP single‐cell isolation platform is not limited to CellSearch‐enriched samples. Lastly, the use of the VyCAP puncher enables a minimal hands‐on time required to isolate single cells with a high throughput.

Finally, the last step of our pipeline is our curated bioinformatics pipeline. This CNV analysis pipeline is applicable to sequencing data of single cells, independent of how the data are acquired and additionally can assess CNVs from comparative genomic hybridization arrays (aCGH) [32, 33]. Our pipeline incorporates a standardized correction of amplification and sequencing biases to the obtained CNV profiles and calls CN alterations as discrete calls rather than exact copy numbers. In our method, each sample is normalized and subsequently corrected using an established control panel to call intrapatient CN alterations resulting in the detection of relative gains and losses. This correction reduces false‐positive calls, which subsequently leads to better calling of true CN alterations. Through our pipeline, the visualization of single CTC CNV profiles with a resolution of 100 kb is possible. In addition to the detection of intrapatient CNV heterogeneity, our method can be built upon to enable additional genomic analysis using the generated WGA products. *Ampli*1™ WGA products also allow for mutation detection using a gene panel designed by Menarini Silicon Biosystems, including oncology‐relevant genes. With this panel, mutations located on amplified fragments, with sufficient distance from *MseI* sites in the genome, can be detected.

Although heterogeneity on a single‐cell level might have clinical value for individual patients in the future, the current value of methods like ours is to serve in basic and translation research to gain more knowledge. Here, we show the feasibility of the detection of heterogeneous copy number alterations in single CTCs although this study is limited to a small number of patients. We envision that single‐cell analysis can contribute to the elucidation of cancer pathophysiology through basic and translational research in larger numbers of patients and CTCs. To enable the routine study of single‐cell genomes, a method which is relatively easy to perform, not too expensive and flexible to apply to different research questions is desirable. Therefore, we designed our pipeline to consist of multiple steps which can be implemented separately or even can be performed at different sites. In addition, we show the feasibility of our pipeline to assess CNVs of single cells after (long term) storage at −80 °C. Conveniently, this facilitates the establishment of single CTC biobanks at research facilities including those collected within clinical trials.

## 5 Conclusion

In conclusion, we have established a single‐cell CNV analysis pipeline and successfully validated this pipeline on patient‐derived CTCs. A major advantage of this pipeline is that it consists of five individual technical steps (CTC enrichment, CTC isolation, whole genome amplification, low‐pass sequencing and bioinformatic analysis) to generate CNV profiles from single cells. This enables modular incorporation of (parts of) the pipeline in current practice for biological and clinical researches. Furthermore, it has a limited hands‐on time and can be routinely applied to patient‐derived CTCs. Finally, our optimized pipeline enables detection of genome‐wide, intrapatient heterogeneity on segments as small as 300 kb. Future research is aimed at incorporating mutation detection into the developed pipeline as well as associating the level of intrapatient heterogeneity with clinical outcome.

## Conflict of interest

The authors declare no conflict of interest.

### Peer Review

The peer review history for this article is available at https://publons.com/publon/10.1002/1878‐0261.13174.

## Author contributions

JWMM conceived of the study, which was developed together with AH, SMW, SS and AMS. PAJM obtained patient samples and clinical data and performed cell enrichment. JK operated the VyCAP puncher and the LSRFortessa. TD, WJCPS and MVD performed the WGA reactions. TD performed all library preparations. EMJB performed all sequencing reactions. The bioinformatics and statistical analyses were performed by TD under supervision of SMW. The manuscript was prepared by TD, PAJM, SWM and AH, and all authors read and approved the manuscript.

## Supporting information


**Fig. S1.** Representative WGA QC scores using the modified VyCAP multiplex PCR. Generated multiplex PCR products were visualized on the MultiNA platform (Shimadzu).

**Fig. S2.** CN profiles of MCF‐7 cells after *Ampli*1 WGA and TruSeq PCR‐free library preparation.

**Fig. S3.** CN profiles after REPLI‐g WGA of MCF‐7 cells.

**Fig. S4.** Density plot depicting all samples with a high number of bins without read counts at a 100kb resolution.

**Fig. S5.** Ten iterations of random downsampling of BC1‐4 at (A) 5 m reads, (B) 2.5 m reads, (C) 1 m reads, and (D) 0.5 m reads; 1 ng of MCF‐7 DNA amplified with Ampli1 at (E) 5 m reads, (F) 2.5 m reads, (G), 1 m reads, and (H) 0.5 m reads; and 1 single MCF‐7 cell at (I) 5 m reads, (J) 2.5 m reads, (K) 1 m reads, and (L) 0.5 m reads.

**Fig. S6.** Density plot of sequence read distribution of mFAST‐SeqS analysis on 20 single cell WGA products. HBD samples (*n* = 12) are indicated in black.

**Fig. S7.** Distribution of mFAST‐SeqS reads in single cell WGA samples.

**Fig. S8.** Examples of clinically relevant CNVs detected by our pipeline.

**Table S1.** List of blacklisted regions by QDNAseq.
**Table S2.** Spearman correlations between ten iterations at different subsampling depths and 10 m + reads of BC1‐4, 1 ng of MCF‐7 DNA amplified with Ampli1, and 1 MCF‐7 cell amplified with Ampli1 (cell C, sample 13).
**Table S3.** Distribution of mFAST‐SeqS reads in single cell WGA samples.
**Table S4.** Spearman correlations between three single MCF‐7 cells amplified with Ampli1 and TruSeq‐prepared libraries and genomic MCF‐7 DNA with a TruSeq‐prepared library.
**Table S5.** Spearman correlations between 27 MCF‐7 strains collected by Ben‐David et al. (2018). Only regions passing the default QDNAseq settings were used.

**Data S1.** R‐markdown file containing the ready‐to‐use R‐script to obtain copy‐number calls, and a heatmap for sequence data (in *.bam format) of single cells.

## Data Availability

The data that support the findings of this study are not publicly available due to privacy or ethical restrictions and are available upon request from the corresponding author j.martens@erasmusmc.nl.
