# Supplementary figures and images for: A pipeline for copy number profiling of single circulating tumour cells to assess intrapatient tumour heterogeneity
Source: Mol Oncol. 2022 Jul 8;16(16):2981–3000. doi: 10.1002/1878-0261.13174 (PMC9394233; doi:10.1002/1878-0261.13174)

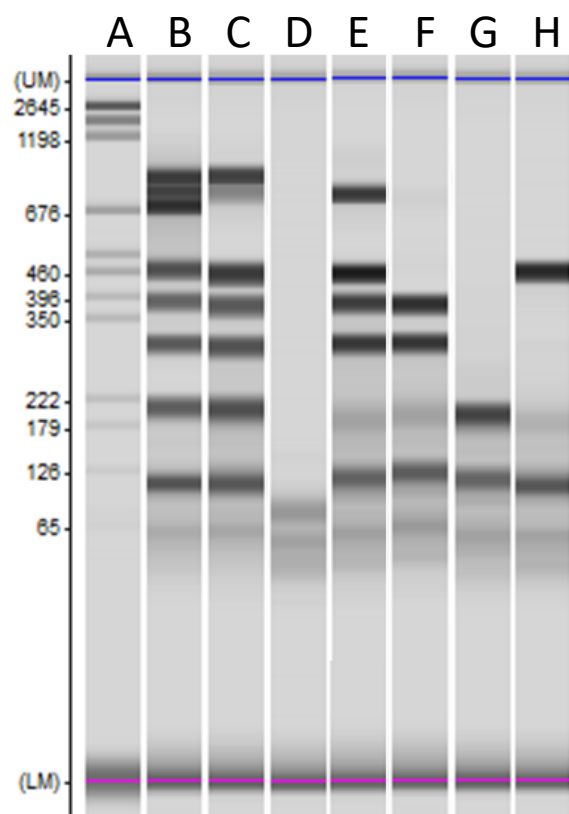

Supplement: Supplementary file 1 — Fig. S1. Representative WGA QC scores using the modified VyCAP multiplex PCR. Generated multiplex PCR products were visualized on the MultiNA platform (Shimadzu). [file MOL2-16-2981-s004.pdf]

A.

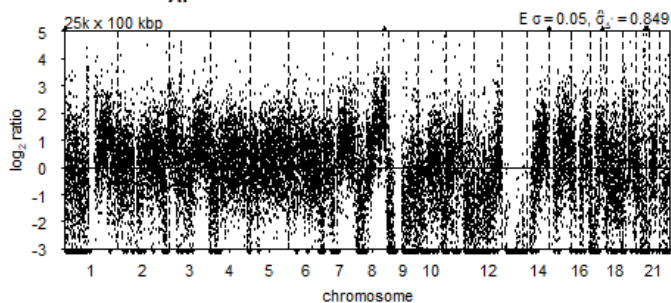

B.

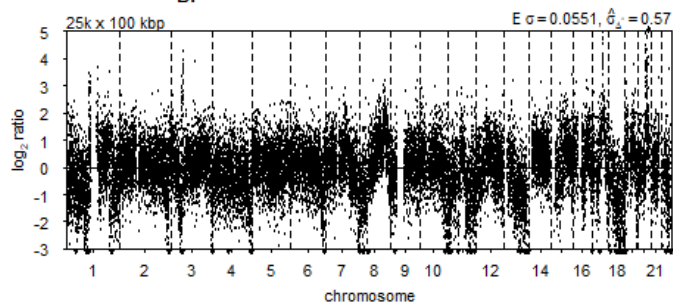

C.

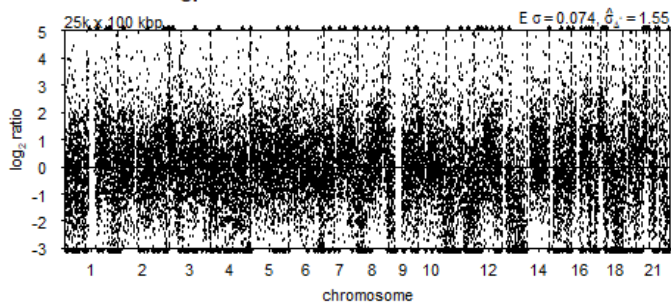

D.

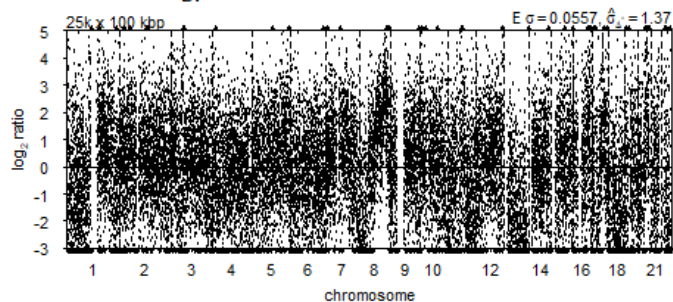

E.

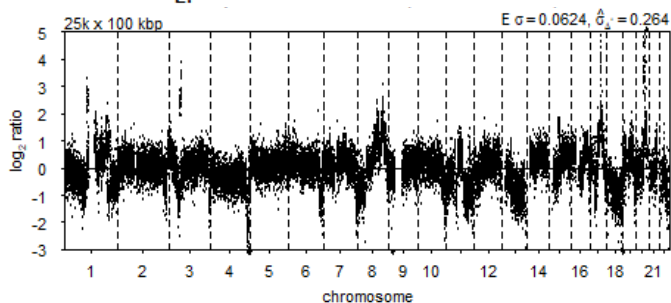

F.

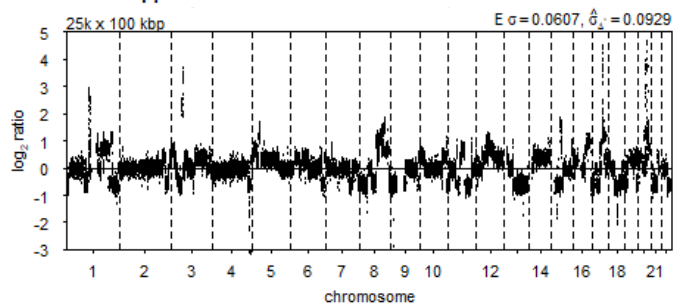

Supplement: Supplementary file 3 — Fig. S3. CN profiles after REPLI‐g WGA of MCF‐7 cells. [file MOL2-16-2981-s006.pdf]

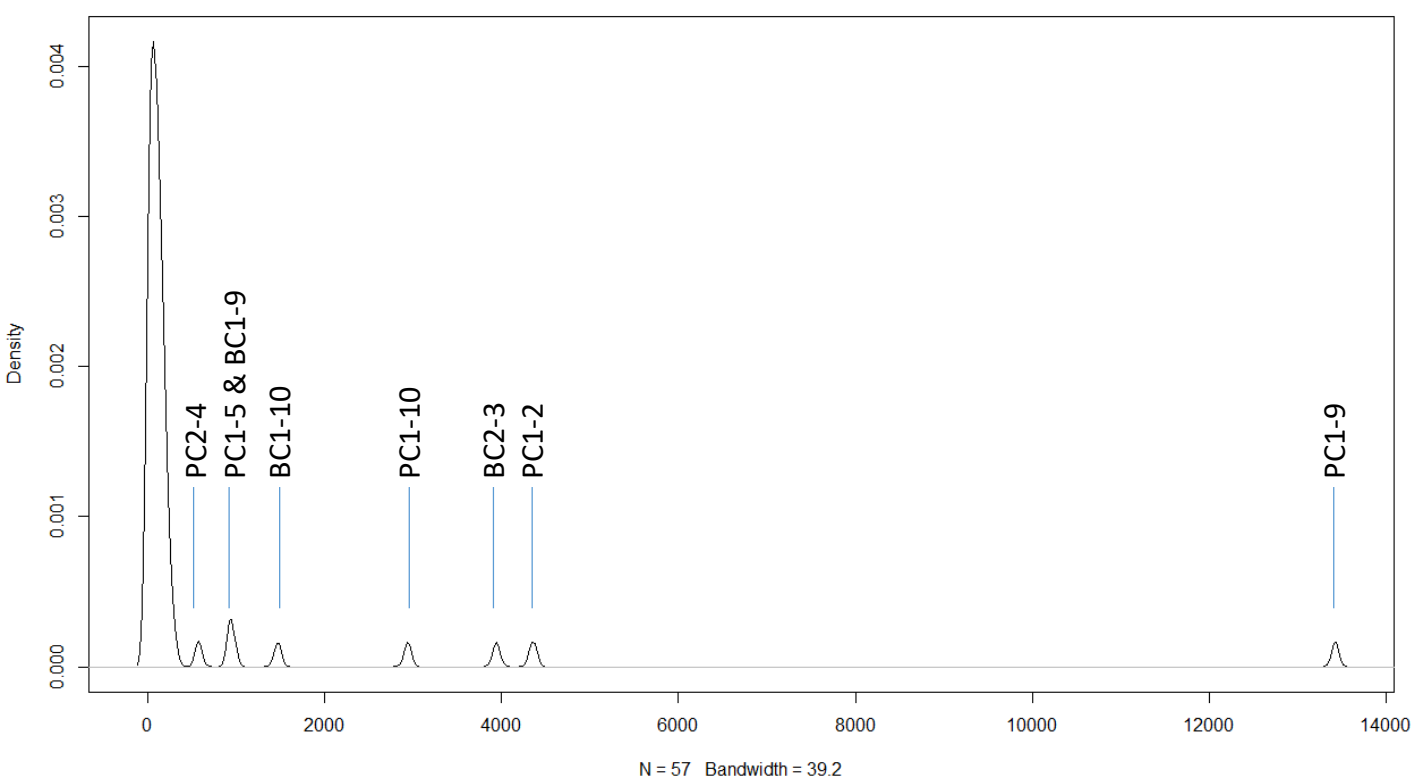

Supplement: Supplementary file 4 — Fig. S4. Density plot depicting all samples with a high number of bins without read counts at a 100kb resolution. [file MOL2-16-2981-s003.pdf]

Densityplot of sequence-read distrubution mFAST-SeqS on single cell WGA

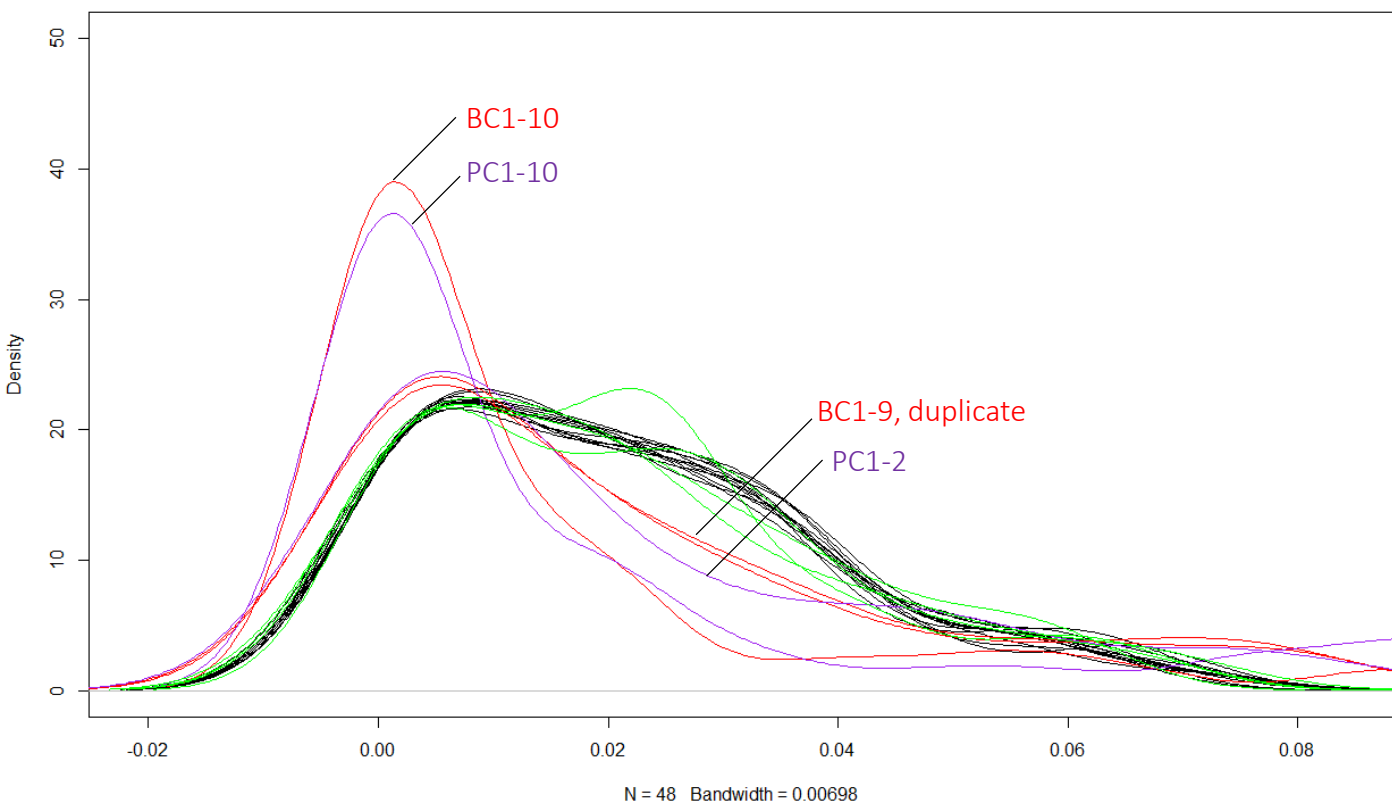

Supplement: Supplementary file 6 — Fig. S6. Density plot of sequence read distribution of mFAST‐SeqS analysis on 20 single cell WGA products. HBD samples (n = 12) are indicated in black. [file MOL2-16-2981-s010.pdf]

A

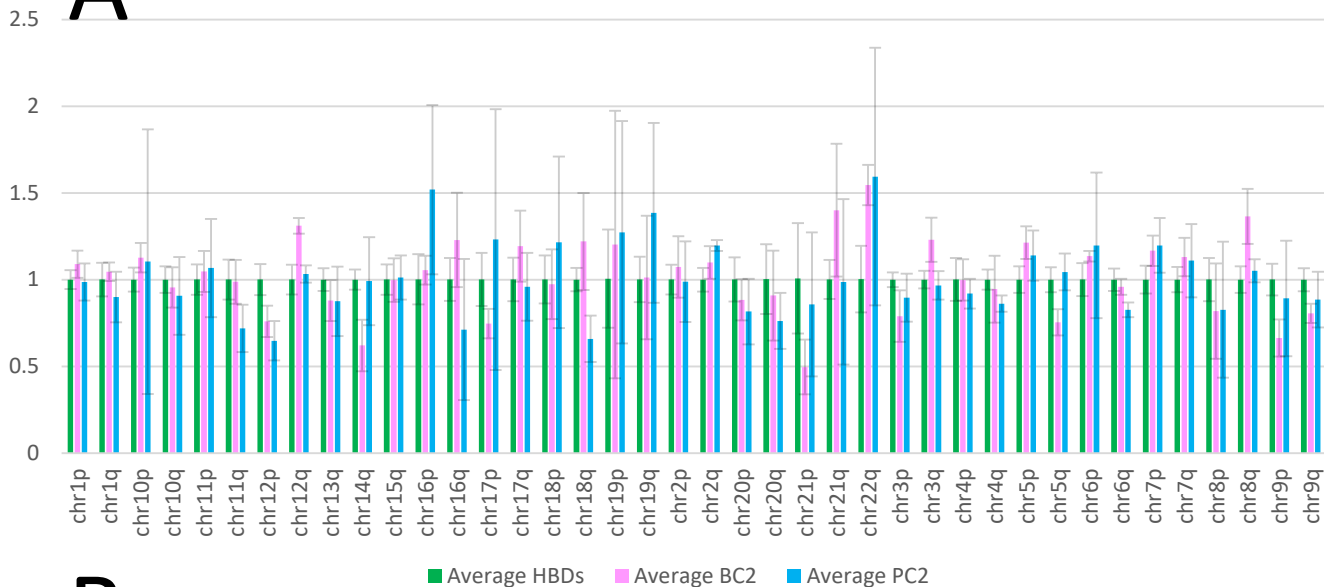

B

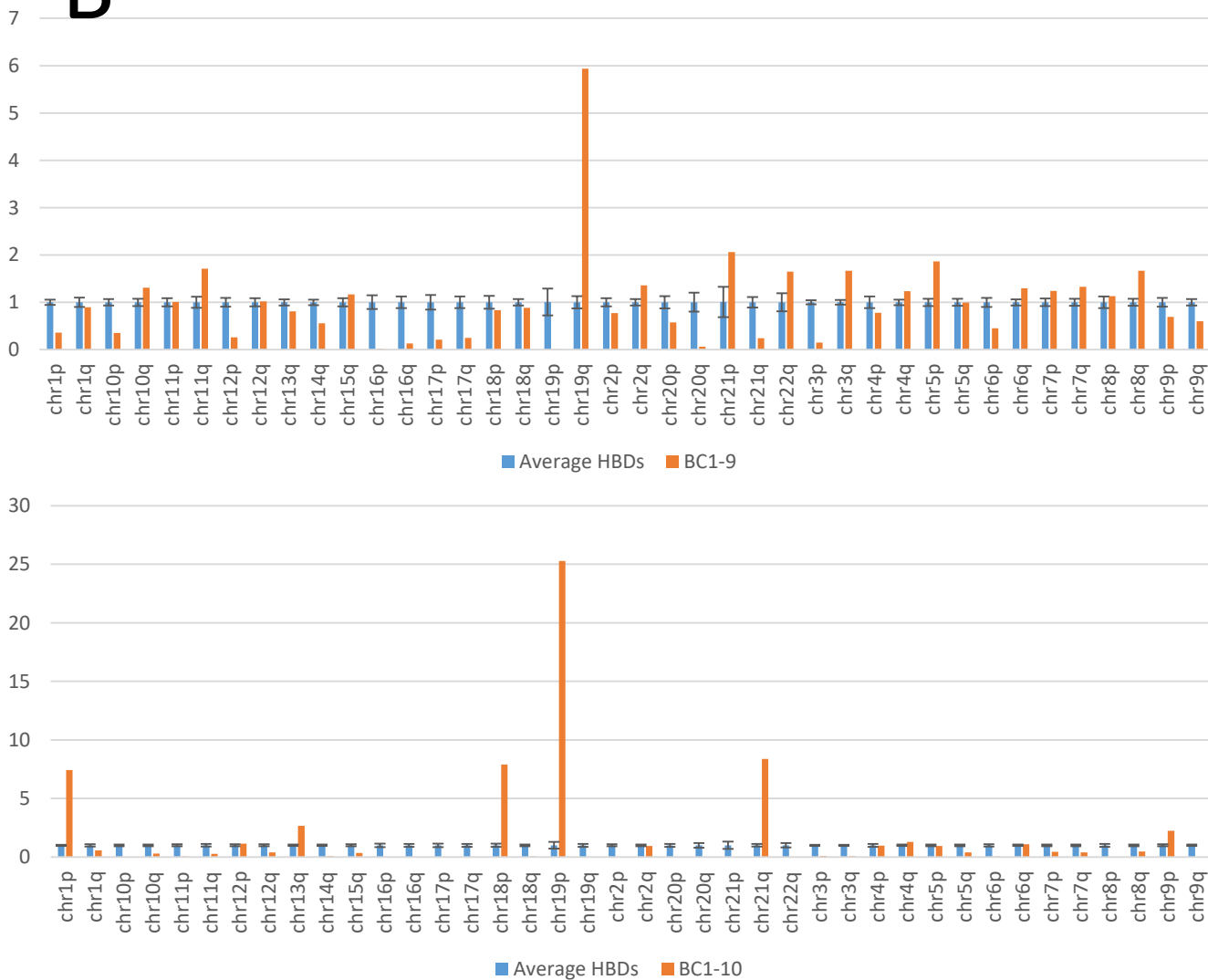

C

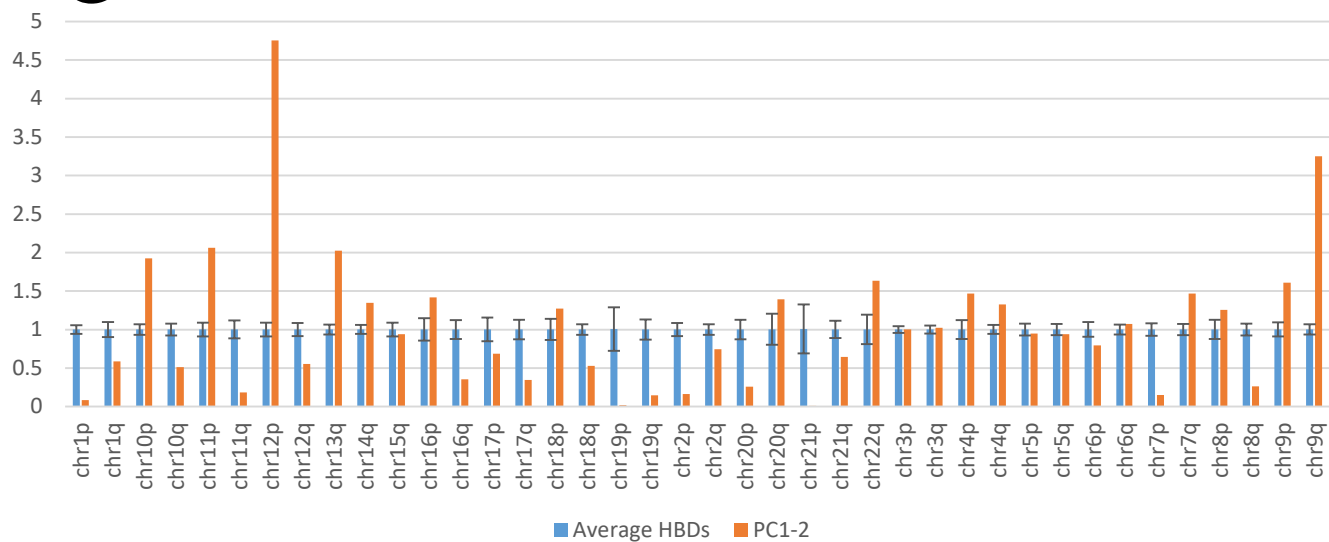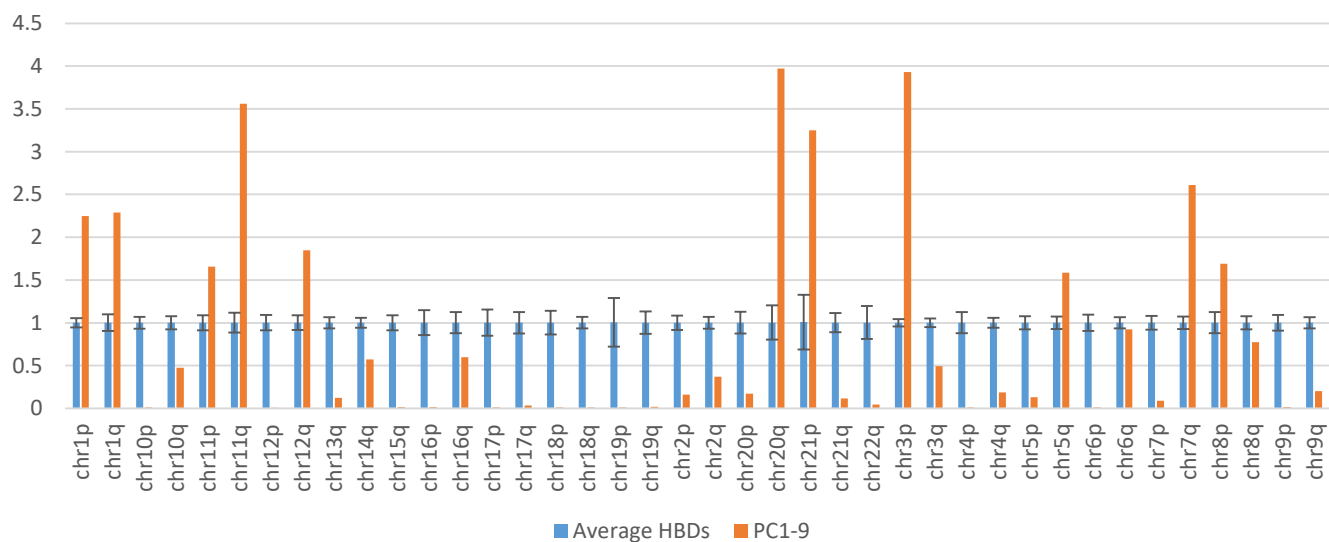

Supplement: Supplementary file 7 — Fig. S7. Distribution of mFAST‐SeqS reads in single cell WGA samples. [file MOL2-16-2981-s001.pdf]

# A

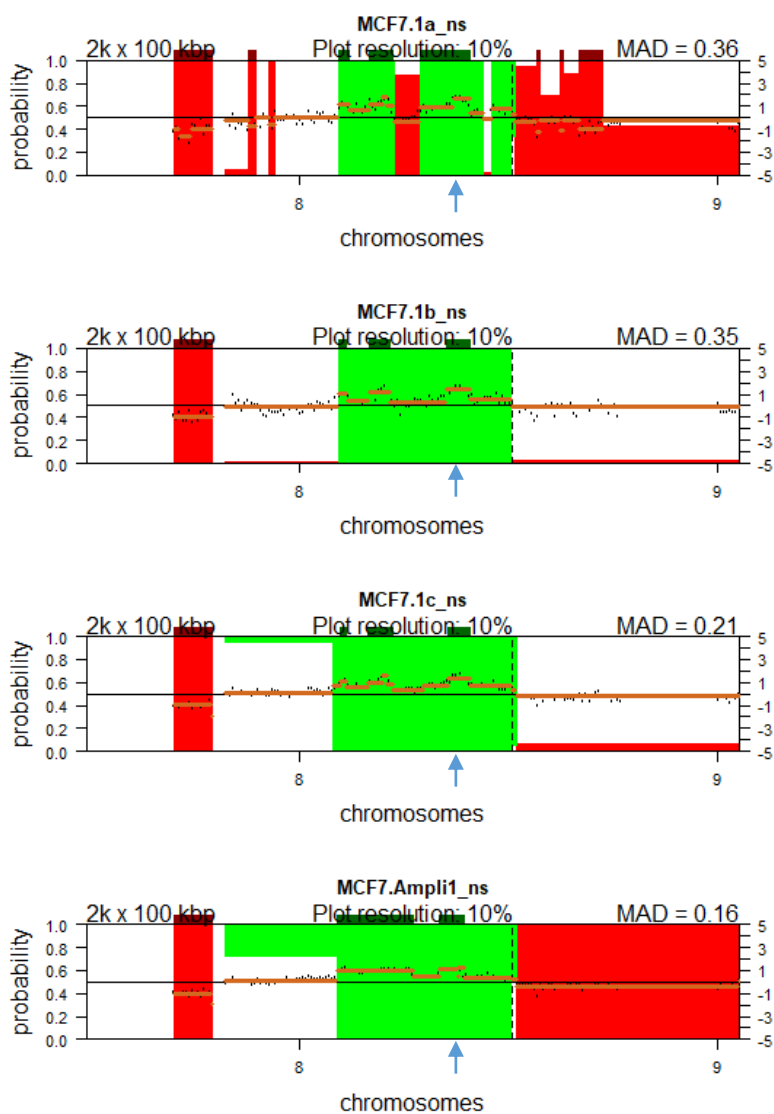

# B

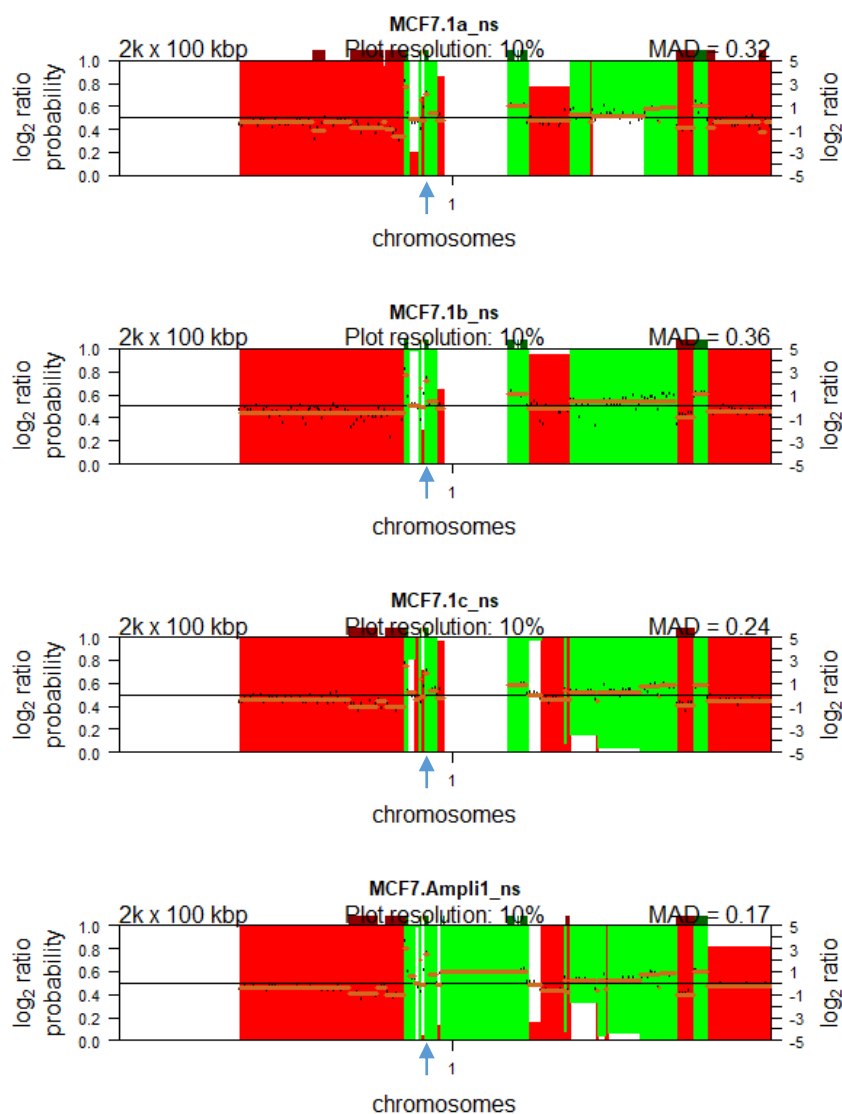

C

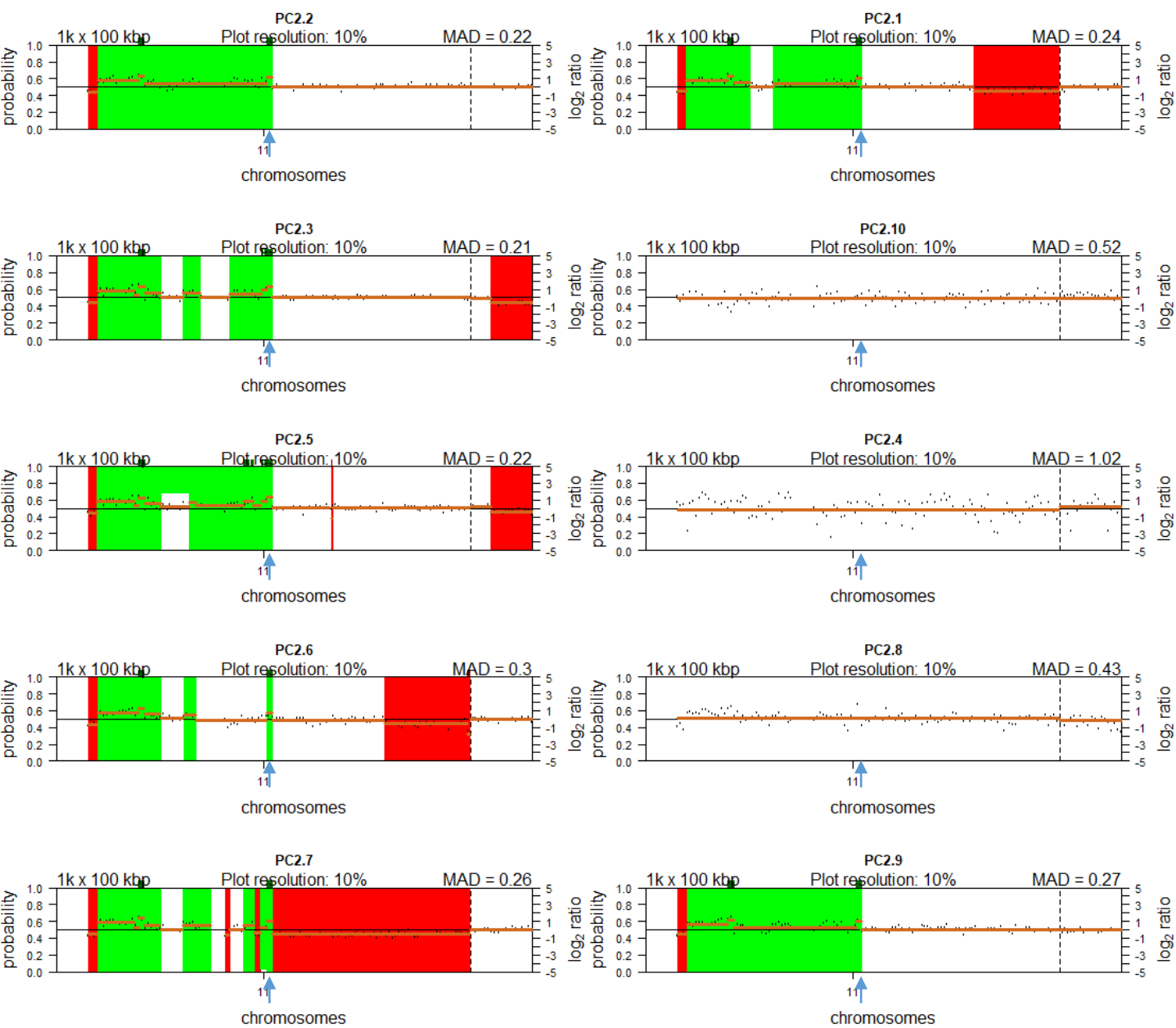

D

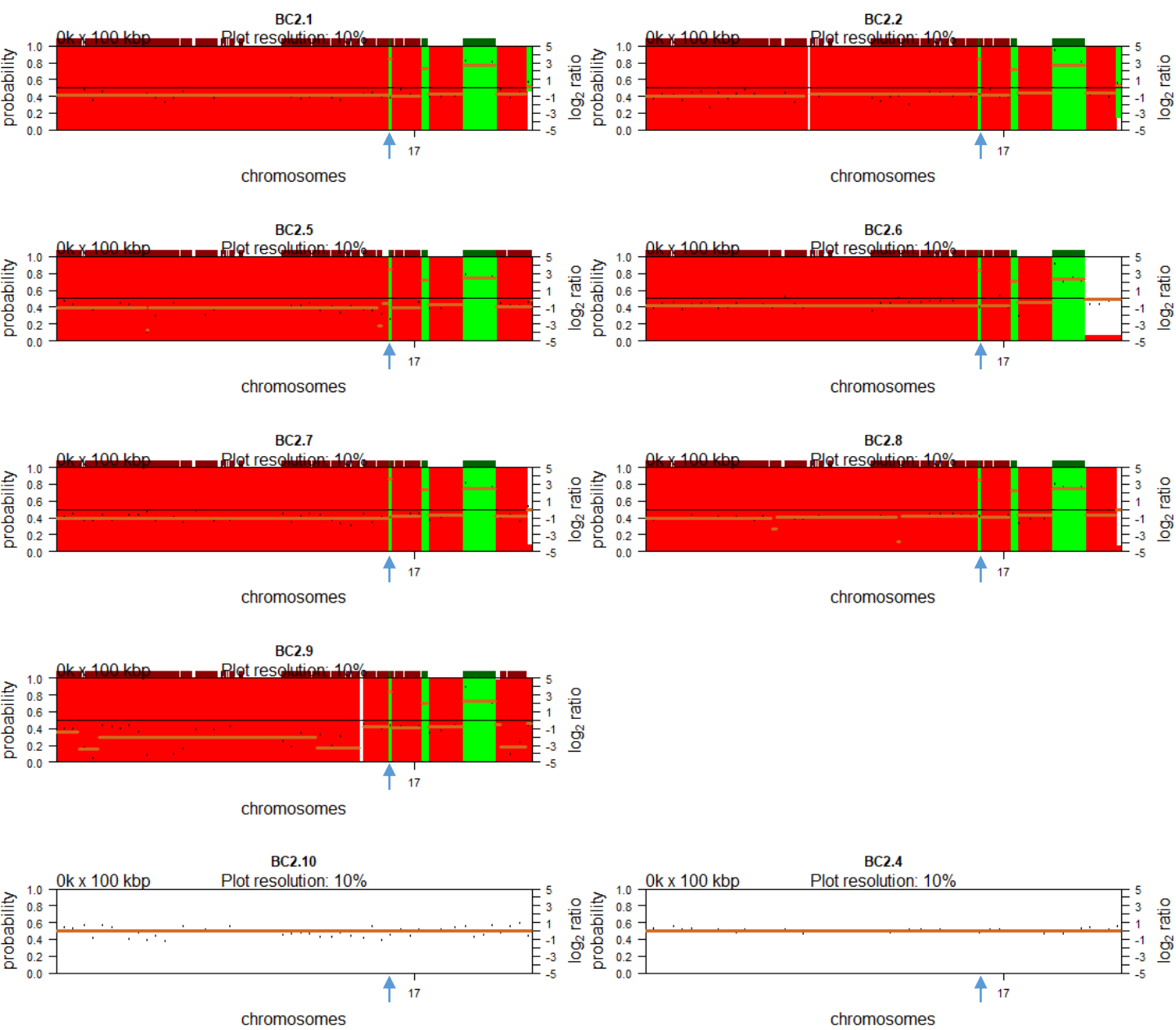

E

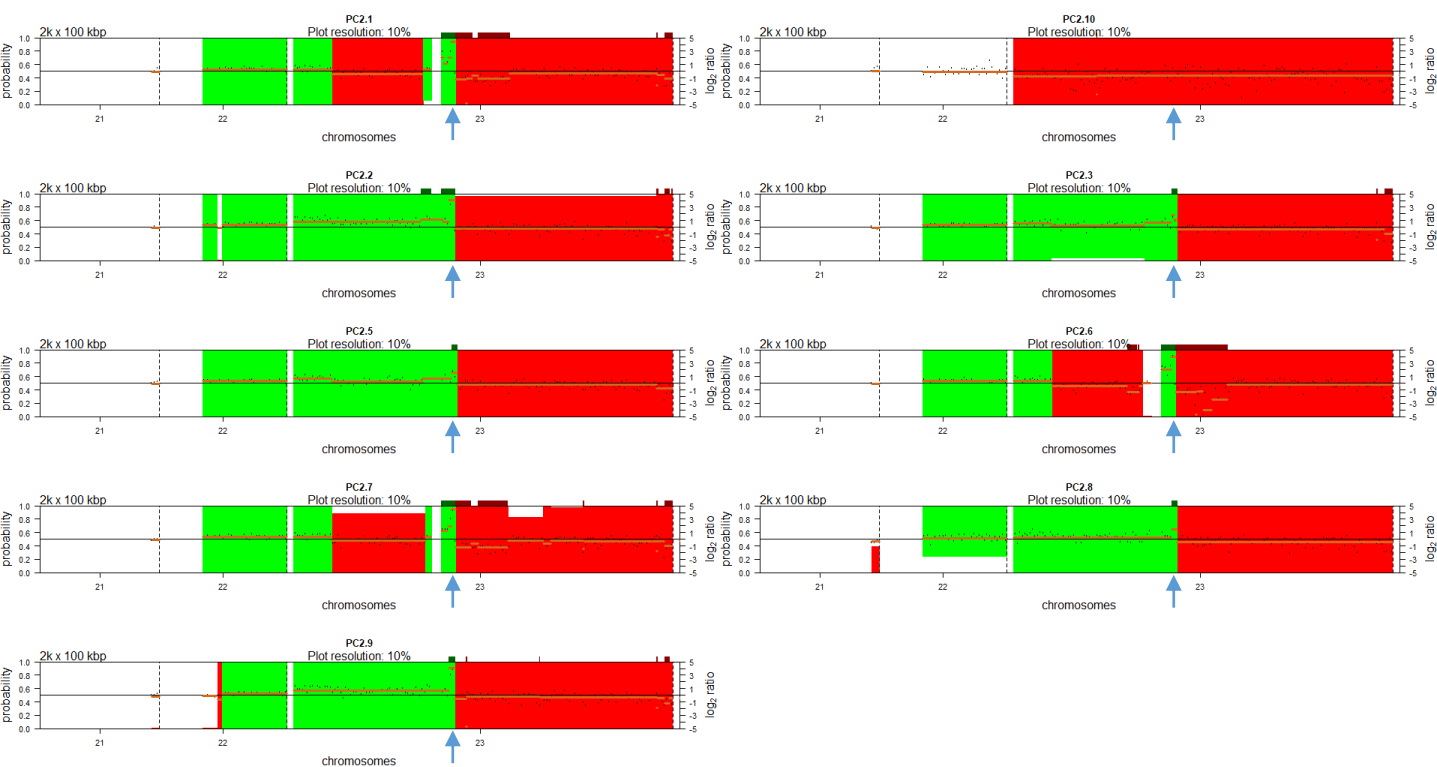

Supplement: Supplementary file 8 — Fig. S8. Examples of clinically relevant CNVs detected by our pipeline. [file MOL2-16-2981-s009.pdf]
