# Supplementary material for: A pipeline for copy number profiling of single circulating tumour cells to assess intrapatient tumour heterogeneity
Source: Mol Oncol. 2022 Jul 8;16(16):2981–3000. doi: 10.1002/1878-0261.13174 (PMC9394233; doi:10.1002/1878-0261.13174)

Single MCF-7, QC-7, sample 11

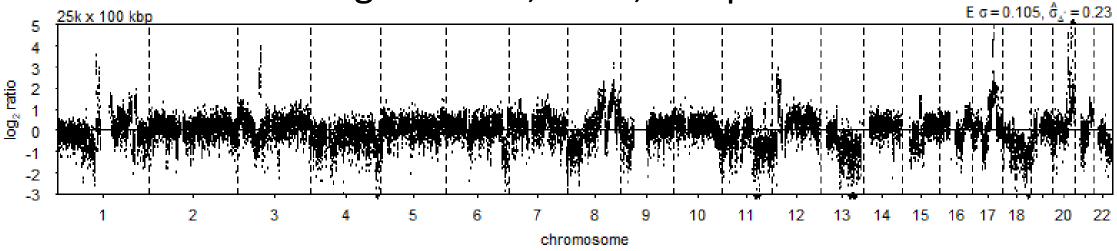

Single MCF-7, QC-7, sample 12

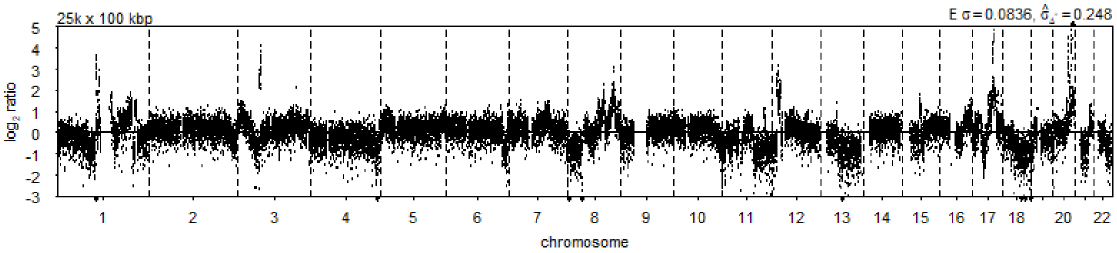

Single MCF-7, QC-7, sample 13

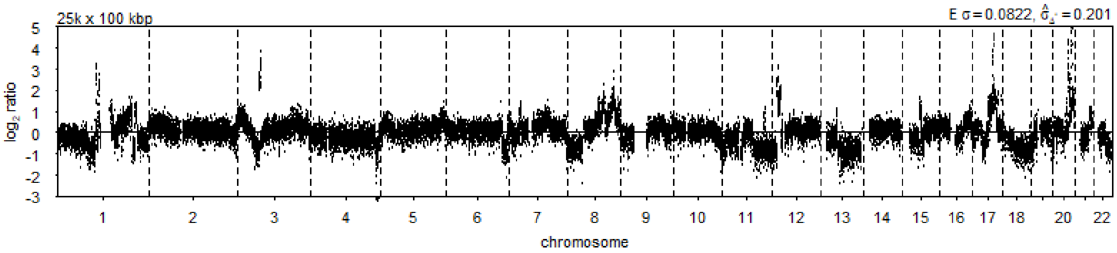

Single MCF-7, QC-6, sample 19

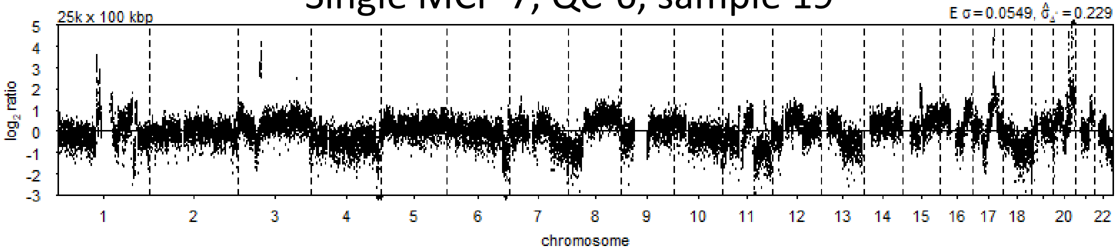

Single MCF-7, QC-5, sample 20

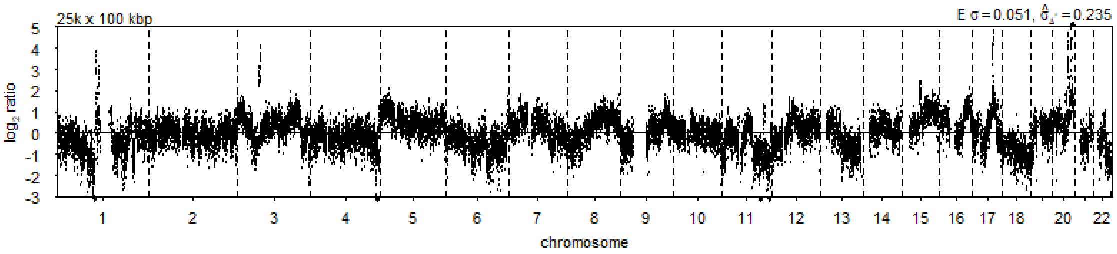

Supplement: Supplementary file 2 — Fig. S2. CN profiles of MCF‐7 cells after Ampli1 WGA and TruSeq PCR‐free library preparation. [file MOL2-16-2981-s005.pdf]
