# Supplementary material for: A pipeline for copy number profiling of single circulating tumour cells to assess intrapatient tumour heterogeneity
Source: Mol Oncol. 2022 Jul 8;16(16):2981–3000. doi: 10.1002/1878-0261.13174 (PMC9394233; doi:10.1002/1878-0261.13174)
Supplement: Supplementary file 5 — Fig. S5. Ten iterations of random downsampling of BC1‐4 at (A) 5 m reads, (B) 2.5 m reads, (C) 1 m reads, and (D) 0.5 m reads; 1 ng of MCF‐7 DNA amplified with Ampli1 at (E) 5 m reads, (F) 2.5 m reads, (G), 1 m reads, and (H) 0.5 m reads; and 1 single MCF‐7 cell at (I) 5 m reads, (J) 2.5 m reads, (K) 1 m reads, and (L) 0.5 m reads. [file MOL2-16-2981-s002.pdf]

A

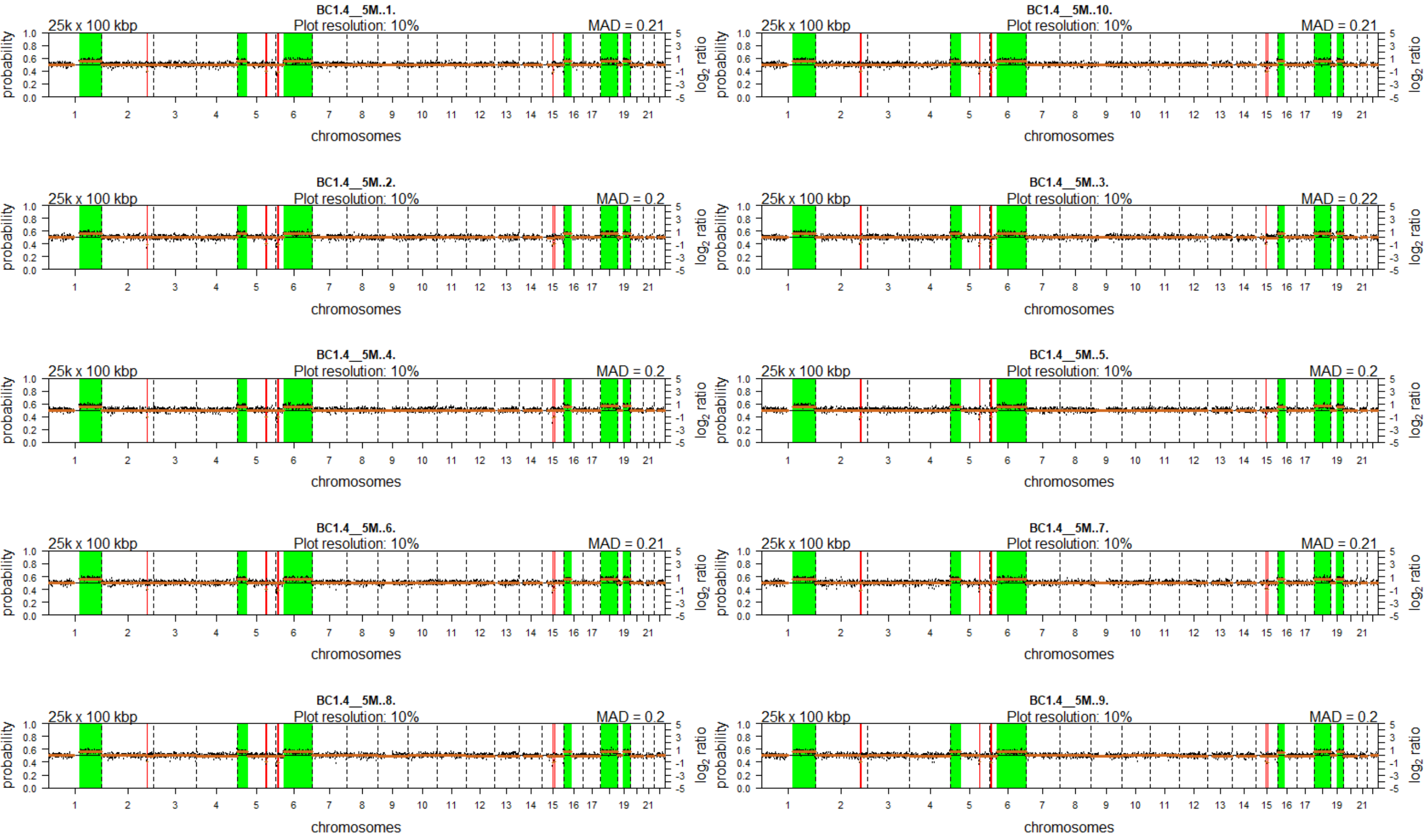
BC1.4__5M..1.
25k x 100 kbp
Plot resolution: 10%
MAD = 0.21
BC1.4__5M..10.
25k x 100 kbp
Plot resolution: 10%
MAD = 0.21
BC1.4__5M..2.
25k x 100 kbp
Plot resolution: 10%
MAD = 0.2
BC1.4__5M..3.
25k x 100 kbp
Plot resolution: 10%
MAD = 0.22
BC1.4__5M..4.
25k x 100 kbp
Plot resolution: 10%
MAD = 0.2
BC1.4__5M..5.
25k x 100 kbp
Plot resolution: 10%
MAD = 0.2
BC1.4__5M..6.
25k x 100 kbp
Plot resolution: 10%
MAD = 0.21
BC1.4__5M..7.
25k x 100 kbp
Plot resolution: 10%
MAD = 0.21
BC1.4__5M..8.
25k x 100 kbp
Plot resolution: 10%
MAD = 0.2
BC1.4__5M..9.
25k x 100 kbp
Plot resolution: 10%
MAD = 0.2
probability
log2 ratio
chromosomes

B

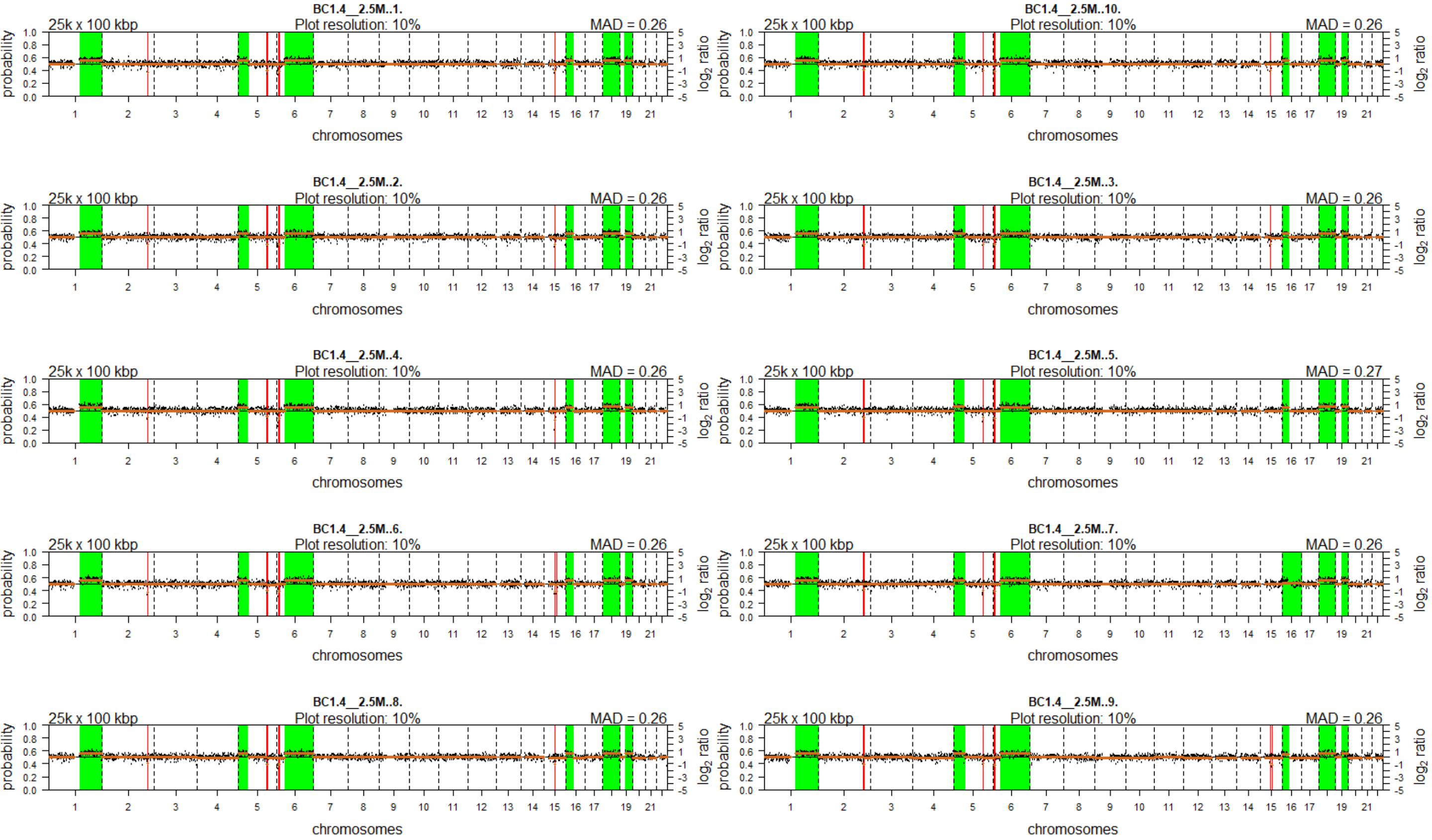
BC1.4__2.5M..1.
25k x 100 kbp
Plot resolution: 10%
MAD = 0.26
probability
log2 ratio
chromosomes
BC1.4__2.5M..10.
25k x 100 kbp
Plot resolution: 10%
MAD = 0.26
BC1.4__2.5M..2.
25k x 100 kbp
Plot resolution: 10%
MAD = 0.26
BC1.4__2.5M..3.
25k x 100 kbp
Plot resolution: 10%
MAD = 0.26
BC1.4__2.5M..4.
25k x 100 kbp
Plot resolution: 10%
MAD = 0.26
BC1.4__2.5M..5.
25k x 100 kbp
Plot resolution: 10%
MAD = 0.27
BC1.4__2.5M..6.
25k x 100 kbp
Plot resolution: 10%
MAD = 0.26
BC1.4__2.5M..7.
25k x 100 kbp
Plot resolution: 10%
MAD = 0.26
BC1.4__2.5M..8.
25k x 100 kbp
Plot resolution: 10%
MAD = 0.26
BC1.4__2.5M..9.
25k x 100 kbp
Plot resolution: 10%
MAD = 0.26

C

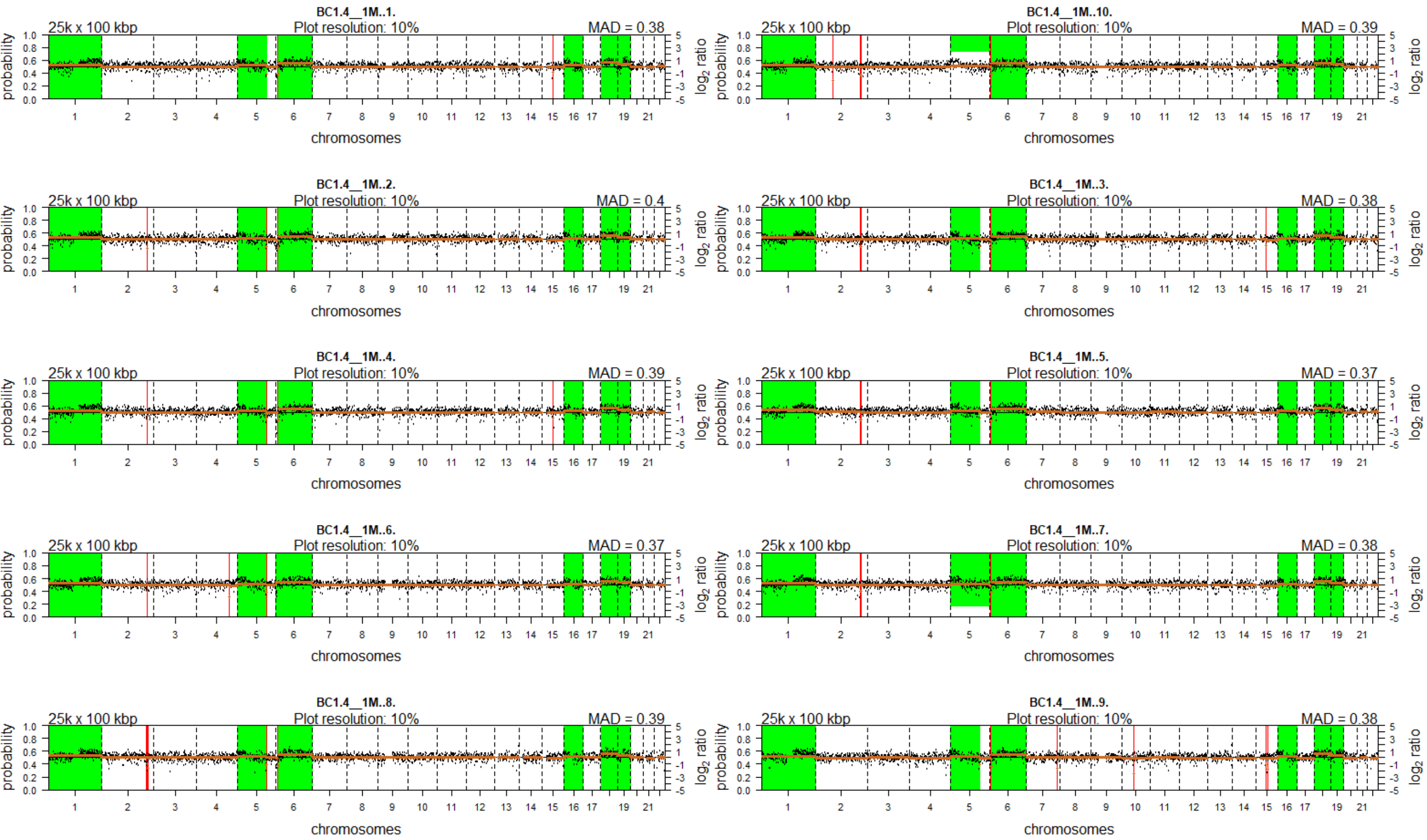
BC1.4__1M..1.
25k x 100 kbp
Plot resolution: 10%
MAD = 0.38
BC1.4__1M..10.
25k x 100 kbp
Plot resolution: 10%
MAD = 0.39
BC1.4__1M..2.
25k x 100 kbp
Plot resolution: 10%
MAD = 0.4
BC1.4__1M..3.
25k x 100 kbp
Plot resolution: 10%
MAD = 0.38
BC1.4__1M..4.
25k x 100 kbp
Plot resolution: 10%
MAD = 0.39
BC1.4__1M..5.
25k x 100 kbp
Plot resolution: 10%
MAD = 0.37
BC1.4__1M..6.
25k x 100 kbp
Plot resolution: 10%
MAD = 0.37
BC1.4__1M..7.
25k x 100 kbp
Plot resolution: 10%
MAD = 0.38
BC1.4__1M..8.
25k x 100 kbp
Plot resolution: 10%
MAD = 0.39
BC1.4__1M..9.
25k x 100 kbp
Plot resolution: 10%
MAD = 0.38
probability
log2 ratio
chromosomes

D

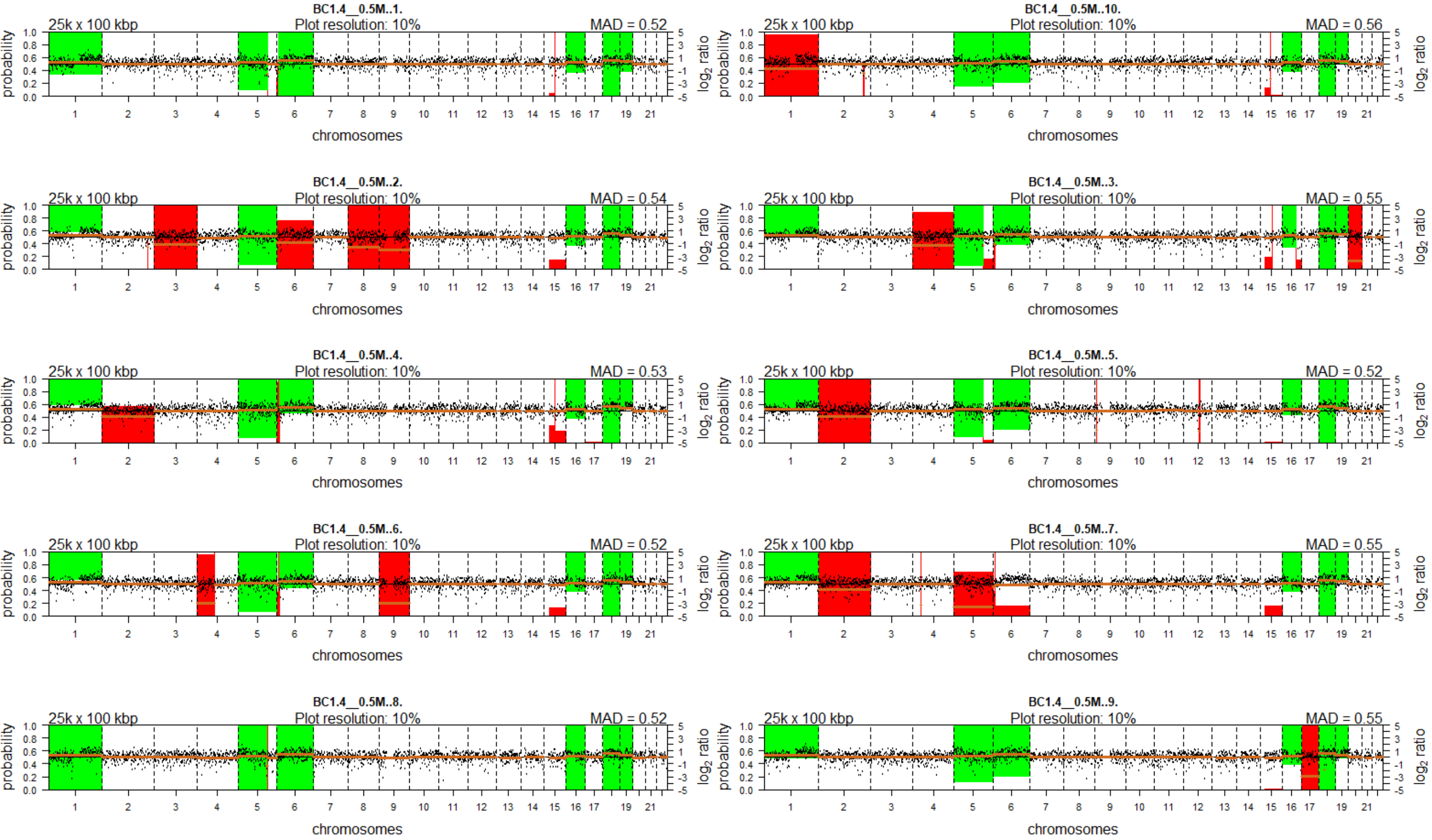
BC1.4__0.5M..1.
25k x 100 kbp
Plot resolution: 10%
MAD = 0.52
BC1.4__0.5M..10.
25k x 100 kbp
Plot resolution: 10%
MAD = 0.56
BC1.4__0.5M..2.
25k x 100 kbp
Plot resolution: 10%
MAD = 0.54
BC1.4__0.5M..3.
25k x 100 kbp
Plot resolution: 10%
MAD = 0.55
BC1.4__0.5M..4.
25k x 100 kbp
Plot resolution: 10%
MAD = 0.53
BC1.4__0.5M..5.
25k x 100 kbp
Plot resolution: 10%
MAD = 0.52
BC1.4__0.5M..6.
25k x 100 kbp
Plot resolution: 10%
MAD = 0.52
BC1.4__0.5M..7.
25k x 100 kbp
Plot resolution: 10%
MAD = 0.55
BC1.4__0.5M..8.
25k x 100 kbp
Plot resolution: 10%
MAD = 0.52
BC1.4__0.5M..9.
25k x 100 kbp
Plot resolution: 10%
MAD = 0.55
probability
log2 ratio
chromosomes

E

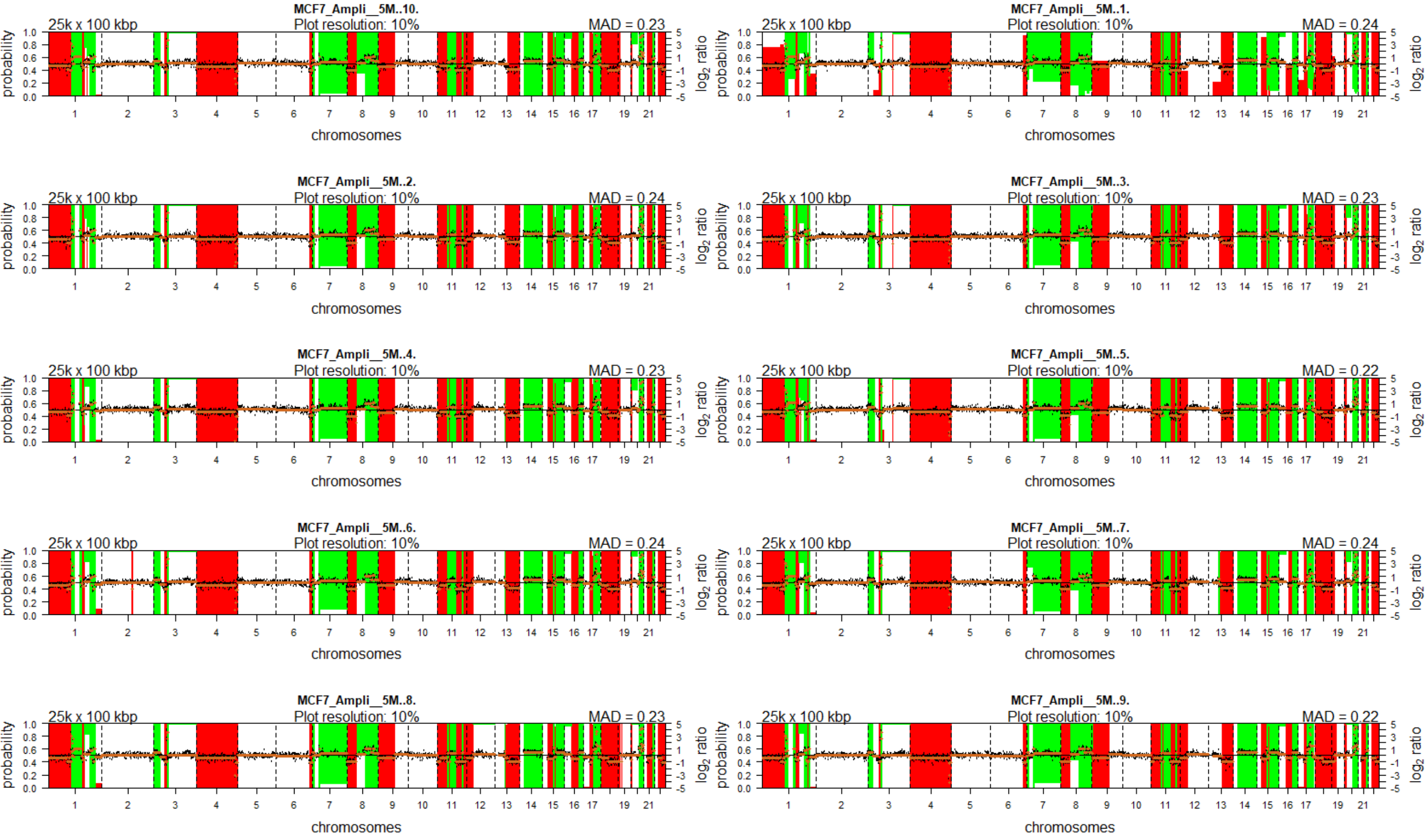
MCF7_Ampli__5M..10.
25k x 100 kbp
Plot resolution: 10%
MAD = 0.23
MCF7_Ampli__5M..1.
25k x 100 kbp
Plot resolution: 10%
MAD = 0.24
MCF7_Ampli__5M..2.
25k x 100 kbp
Plot resolution: 10%
MAD = 0.24
MCF7_Ampli__5M..3.
25k x 100 kbp
Plot resolution: 10%
MAD = 0.23
MCF7_Ampli__5M..4.
25k x 100 kbp
Plot resolution: 10%
MAD = 0.23
MCF7_Ampli__5M..5.
25k x 100 kbp
Plot resolution: 10%
MAD = 0.22
MCF7_Ampli__5M..6.
25k x 100 kbp
Plot resolution: 10%
MAD = 0.24
MCF7_Ampli__5M..7.
25k x 100 kbp
Plot resolution: 10%
MAD = 0.24
MCF7_Ampli__5M..8.
25k x 100 kbp
Plot resolution: 10%
MAD = 0.23
MCF7_Ampli__5M..9.
25k x 100 kbp
Plot resolution: 10%
MAD = 0.22
probability
log2 ratio
chromosomes

F

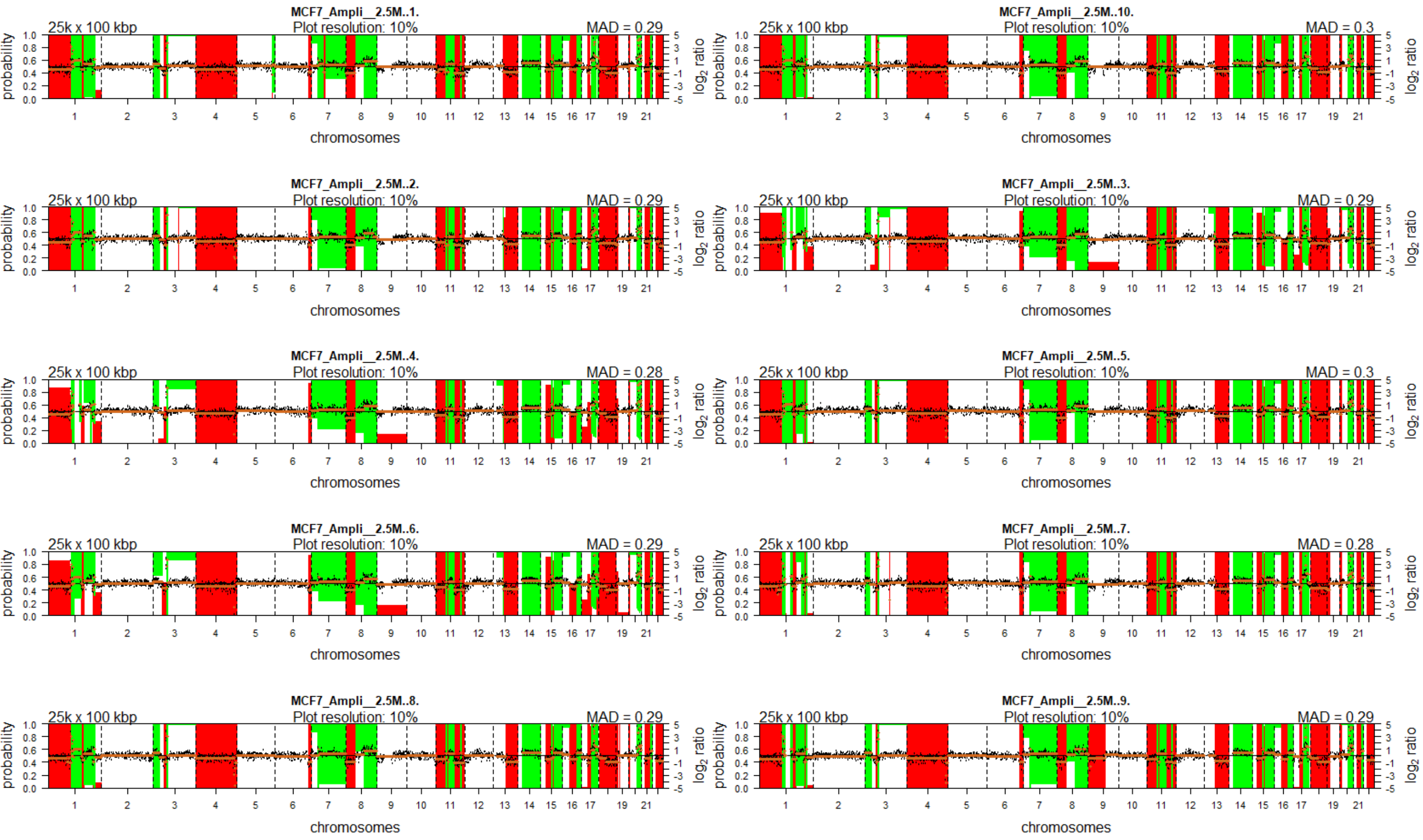
MCF7_Ampli__2.5M..1.
25k x 100 kbp
Plot resolution: 10%
MAD = 0.29
MCF7_Ampli__2.5M..10.
25k x 100 kbp
Plot resolution: 10%
MAD = 0.3
MCF7_Ampli__2.5M..2.
25k x 100 kbp
Plot resolution: 10%
MAD = 0.29
MCF7_Ampli__2.5M..3.
25k x 100 kbp
Plot resolution: 10%
MAD = 0.29
MCF7_Ampli__2.5M..4.
25k x 100 kbp
Plot resolution: 10%
MAD = 0.28
MCF7_Ampli__2.5M..5.
25k x 100 kbp
Plot resolution: 10%
MAD = 0.3
MCF7_Ampli__2.5M..6.
25k x 100 kbp
Plot resolution: 10%
MAD = 0.29
MCF7_Ampli__2.5M..7.
25k x 100 kbp
Plot resolution: 10%
MAD = 0.28
MCF7_Ampli__2.5M..8.
25k x 100 kbp
Plot resolution: 10%
MAD = 0.29
MCF7_Ampli__2.5M..9.
25k x 100 kbp
Plot resolution: 10%
MAD = 0.29
probability
log2 ratio
chromosomes

G

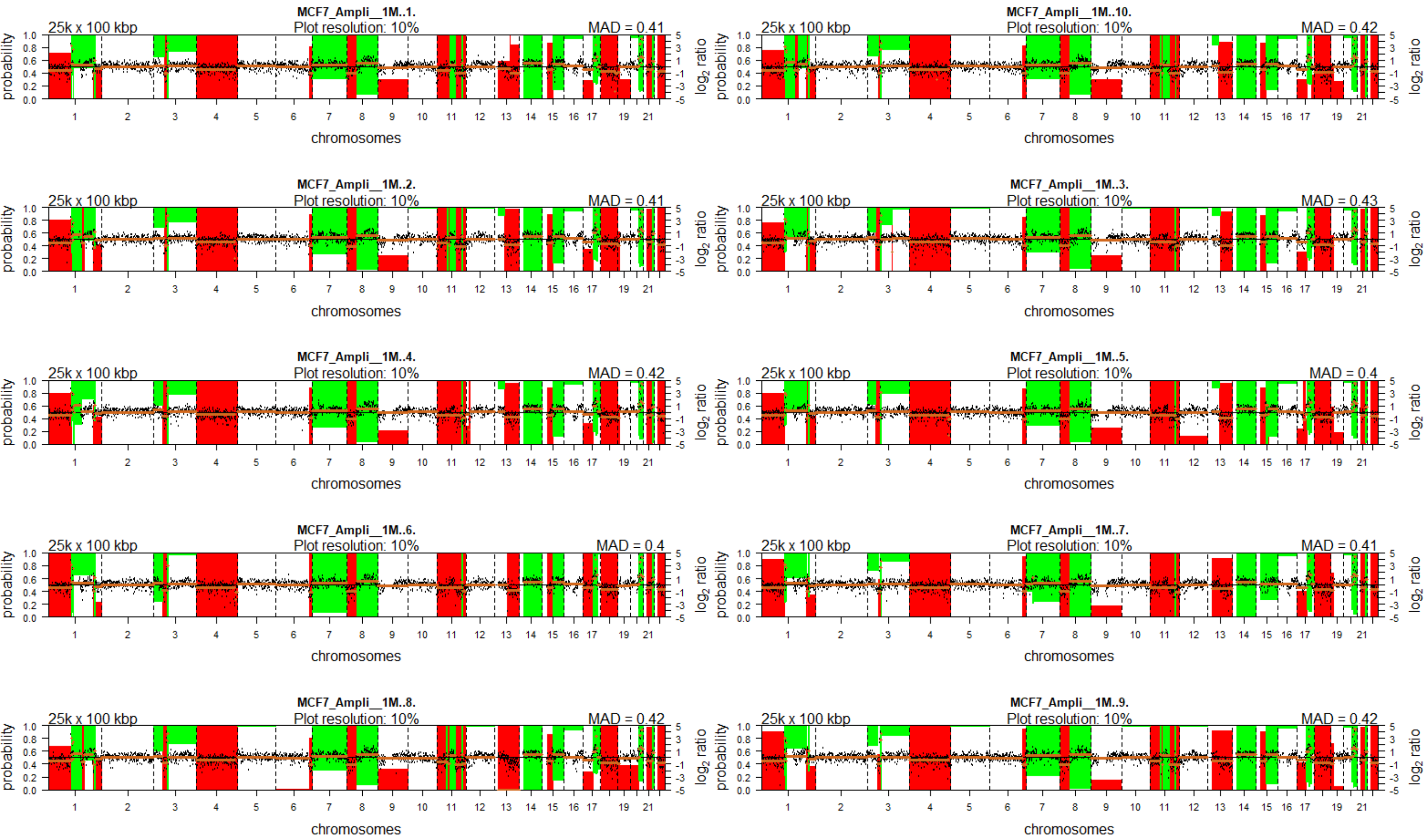
MCF7_Ampli__1M..1.
25k x 100 kbp
Plot resolution: 10%
MAD = 0.41
MCF7_Ampli__1M..10.
25k x 100 kbp
Plot resolution: 10%
MAD = 0.42
MCF7_Ampli__1M..2.
25k x 100 kbp
Plot resolution: 10%
MAD = 0.41
MCF7_Ampli__1M..3.
25k x 100 kbp
Plot resolution: 10%
MAD = 0.43
MCF7_Ampli__1M..4.
25k x 100 kbp
Plot resolution: 10%
MAD = 0.42
MCF7_Ampli__1M..5.
25k x 100 kbp
Plot resolution: 10%
MAD = 0.4
MCF7_Ampli__1M..6.
25k x 100 kbp
Plot resolution: 10%
MAD = 0.4
MCF7_Ampli__1M..7.
25k x 100 kbp
Plot resolution: 10%
MAD = 0.41
MCF7_Ampli__1M..8.
25k x 100 kbp
Plot resolution: 10%
MAD = 0.42
MCF7_Ampli__1M..9.
25k x 100 kbp
Plot resolution: 10%
MAD = 0.42
probability
log2 ratio
chromosomes

H

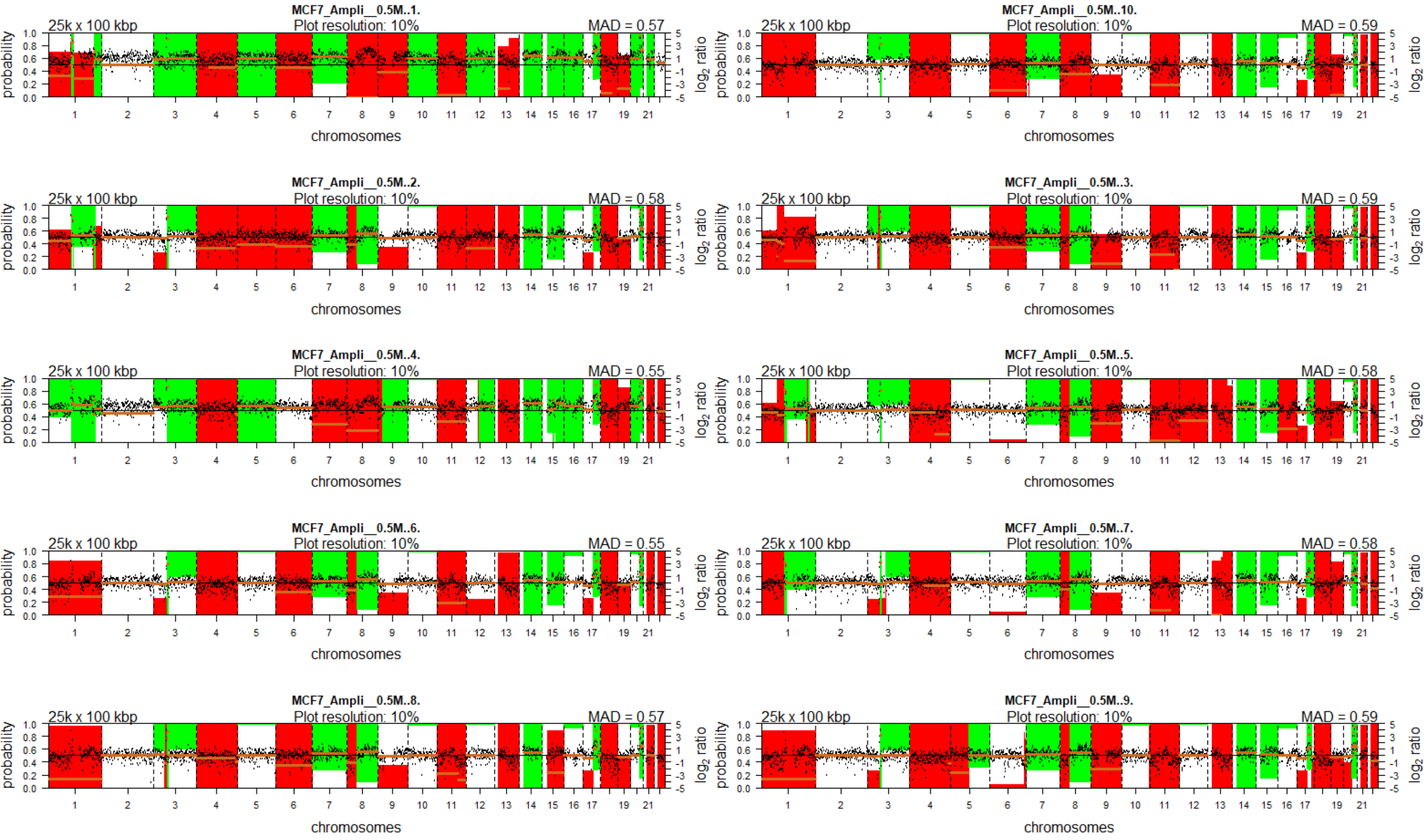
MCF7_Ampli__0.5M..1.
25k x 100 kbp
Plot resolution: 10%
MAD = 0.57
MCF7_Ampli__0.5M..10.
25k x 100 kbp
Plot resolution: 10%
MAD = 0.59
MCF7_Ampli__0.5M..2.
25k x 100 kbp
Plot resolution: 10%
MAD = 0.58
MCF7_Ampli__0.5M..3.
25k x 100 kbp
Plot resolution: 10%
MAD = 0.59
MCF7_Ampli__0.5M..4.
25k x 100 kbp
Plot resolution: 10%
MAD = 0.55
MCF7_Ampli__0.5M..5.
25k x 100 kbp
Plot resolution: 10%
MAD = 0.58
MCF7_Ampli__0.5M..6.
25k x 100 kbp
Plot resolution: 10%
MAD = 0.55
MCF7_Ampli__0.5M..7.
25k x 100 kbp
Plot resolution: 10%
MAD = 0.58
MCF7_Ampli__0.5M..8.
25k x 100 kbp
Plot resolution: 10%
MAD = 0.57
MCF7_Ampli__0.5M..9.
25k x 100 kbp
Plot resolution: 10%
MAD = 0.59
probability
log2 ratio
chromosomes

I

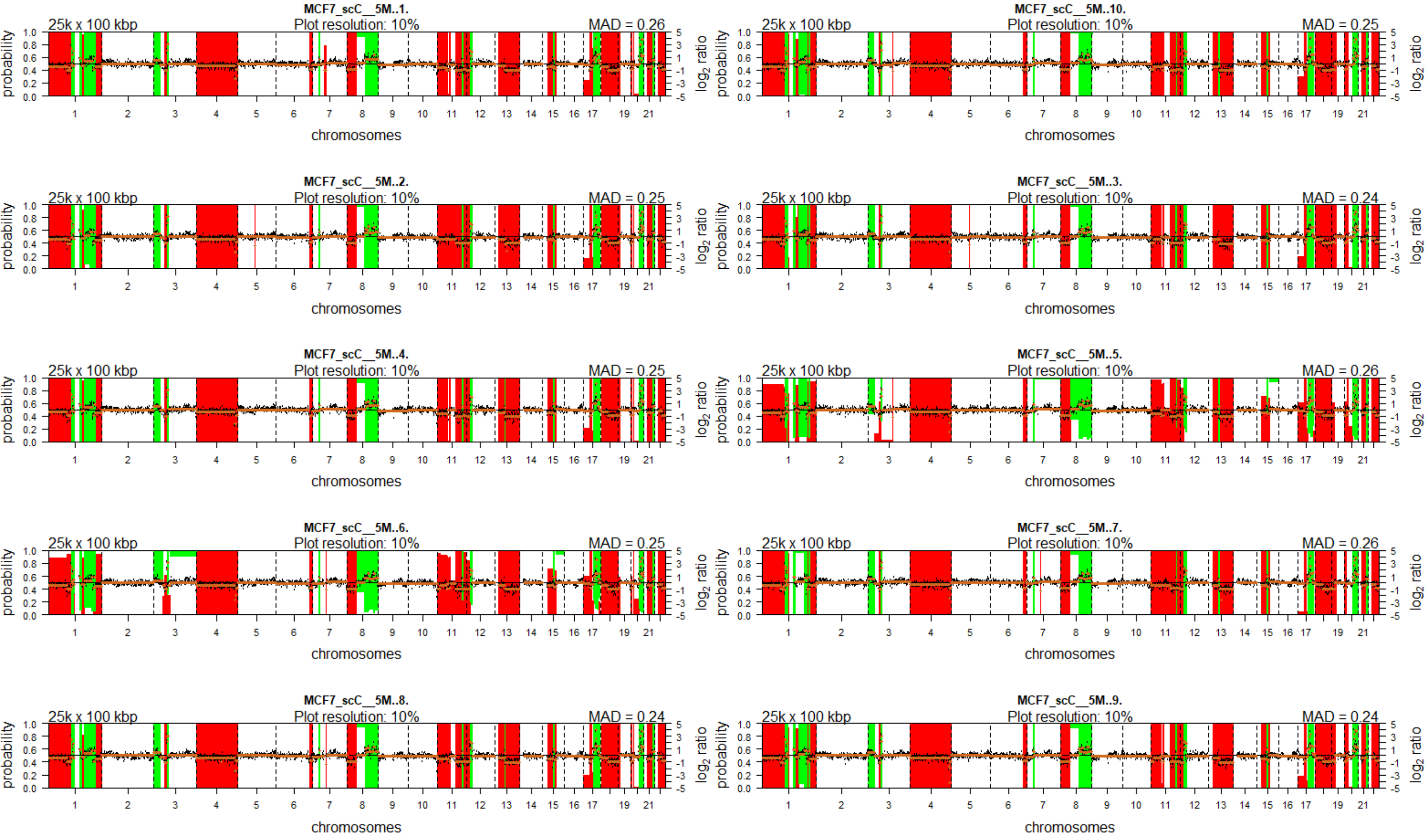
MCF7_scC__5M..1.
25k x 100 kbp
Plot resolution: 10%
MAD = 0.26
MCF7_scC__5M..10.
25k x 100 kbp
Plot resolution: 10%
MAD = 0.25
MCF7_scC__5M..2.
25k x 100 kbp
Plot resolution: 10%
MAD = 0.25
MCF7_scC__5M..3.
25k x 100 kbp
Plot resolution: 10%
MAD = 0.24
MCF7_scC__5M..4.
25k x 100 kbp
Plot resolution: 10%
MAD = 0.25
MCF7_scC__5M..5.
25k x 100 kbp
Plot resolution: 10%
MAD = 0.26
MCF7_scC__5M..6.
25k x 100 kbp
Plot resolution: 10%
MAD = 0.25
MCF7_scC__5M..7.
25k x 100 kbp
Plot resolution: 10%
MAD = 0.26
MCF7_scC__5M..8.
25k x 100 kbp
Plot resolution: 10%
MAD = 0.24
MCF7_scC__5M..9.
25k x 100 kbp
Plot resolution: 10%
MAD = 0.24
probability
log2 ratio
chromosomes

J

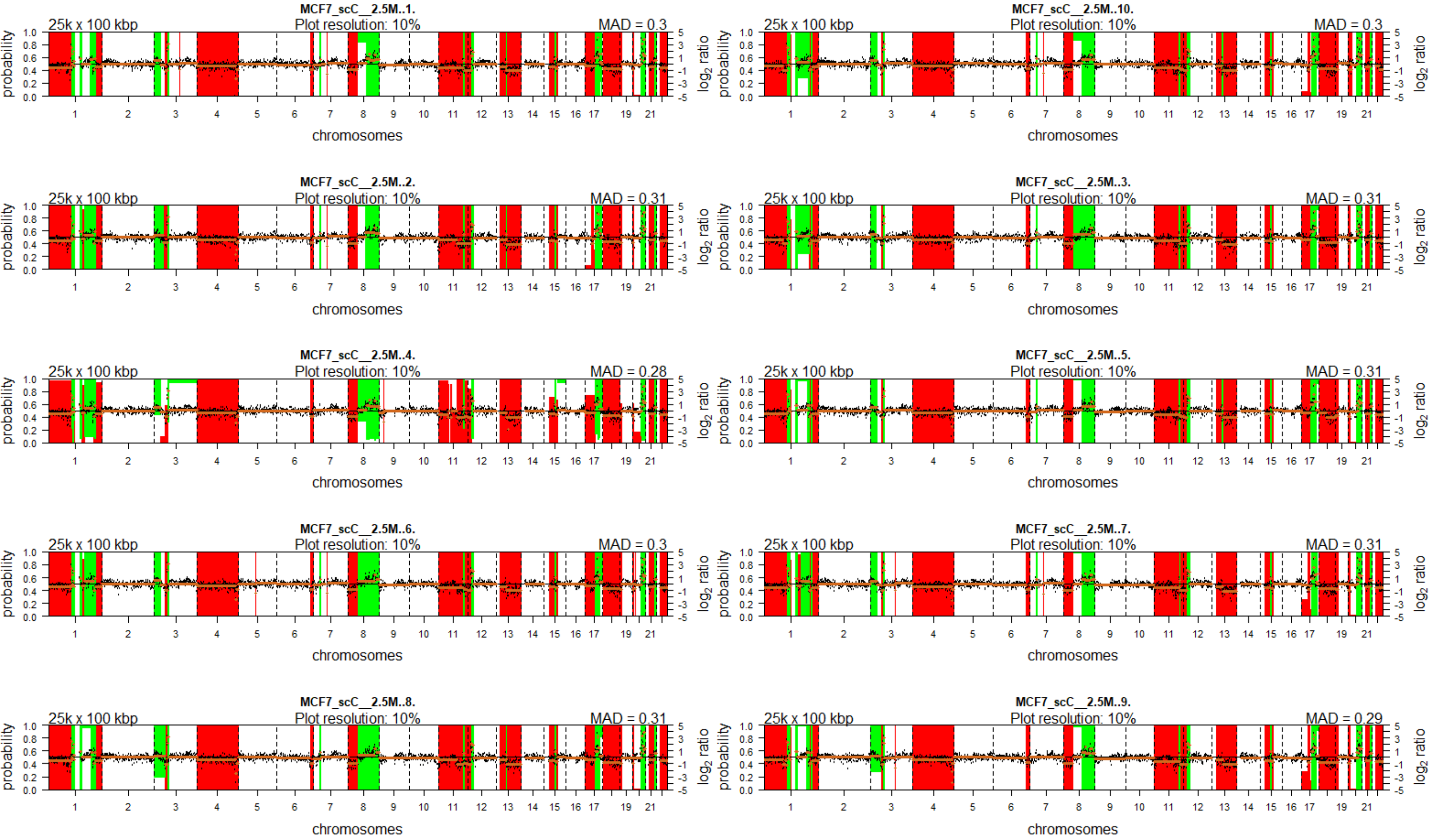
MCF7_scC__2.5M..1.
MCF7_scC__2.5M..10.
MCF7_scC__2.5M..2.
MCF7_scC__2.5M..3.
MCF7_scC__2.5M..4.
MCF7_scC__2.5M..5.
MCF7_scC__2.5M..6.
MCF7_scC__2.5M..7.
MCF7_scC__2.5M..8.
MCF7_scC__2.5M..9.
25k x 100 kbp
Plot resolution: 10%
MAD = 0.3
MAD = 0.31
MAD = 0.28
MAD = 0.29
probability
log2 ratio
chromosomes

K

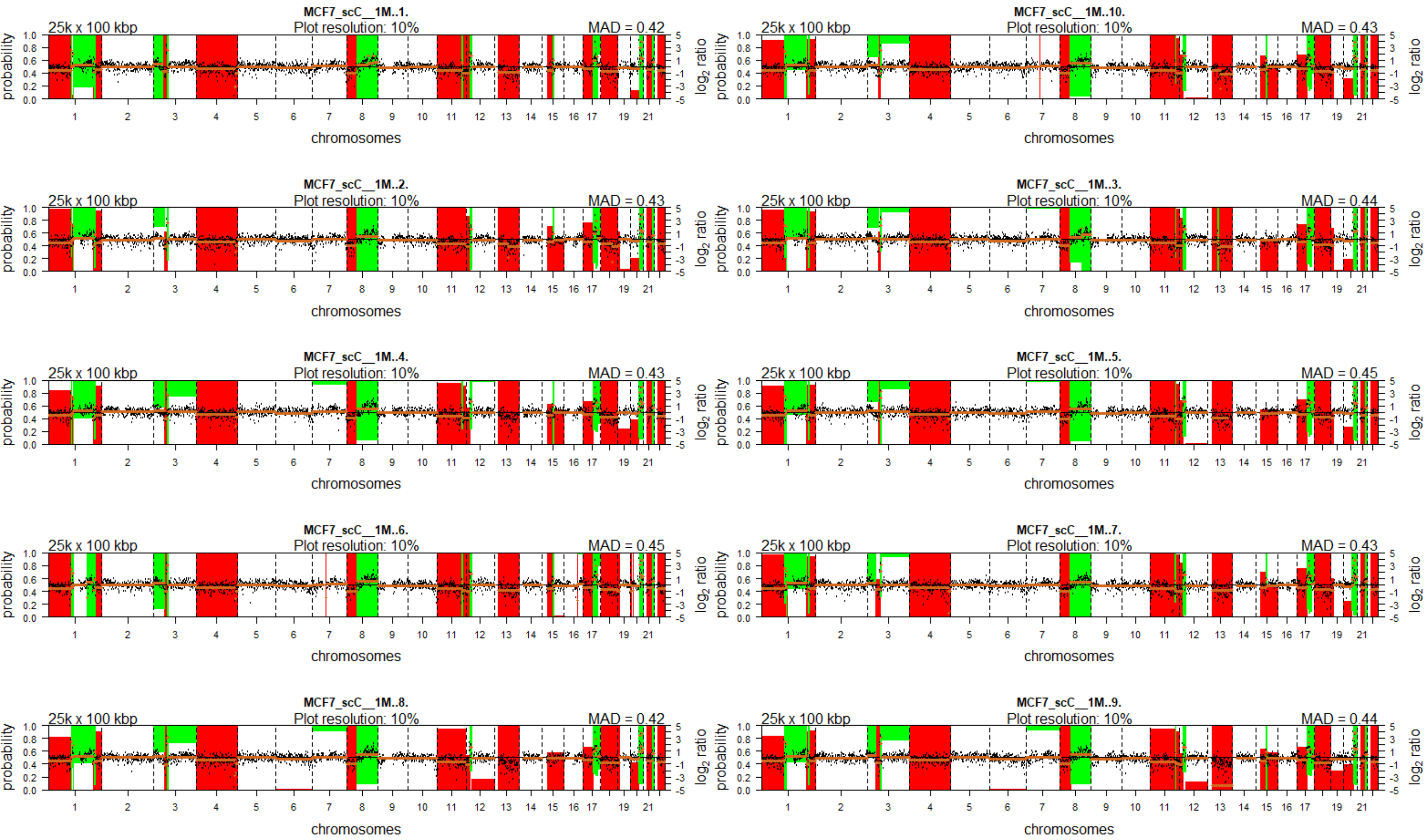
MCF7_scC__1M..1.
25k x 100 kbp
Plot resolution: 10%
MAD = 0.42
MCF7_scC__1M..10.
MAD = 0.43
MCF7_scC__1M..2.
MAD = 0.43
MCF7_scC__1M..3.
MAD = 0.44
MCF7_scC__1M..4.
MAD = 0.43
MCF7_scC__1M..5.
MAD = 0.45
MCF7_scC__1M..6.
MAD = 0.45
MCF7_scC__1M..7.
MAD = 0.43
MCF7_scC__1M..8.
MAD = 0.42
MCF7_scC__1M..9.
MAD = 0.44
probability
log2 ratio
chromosomes

L

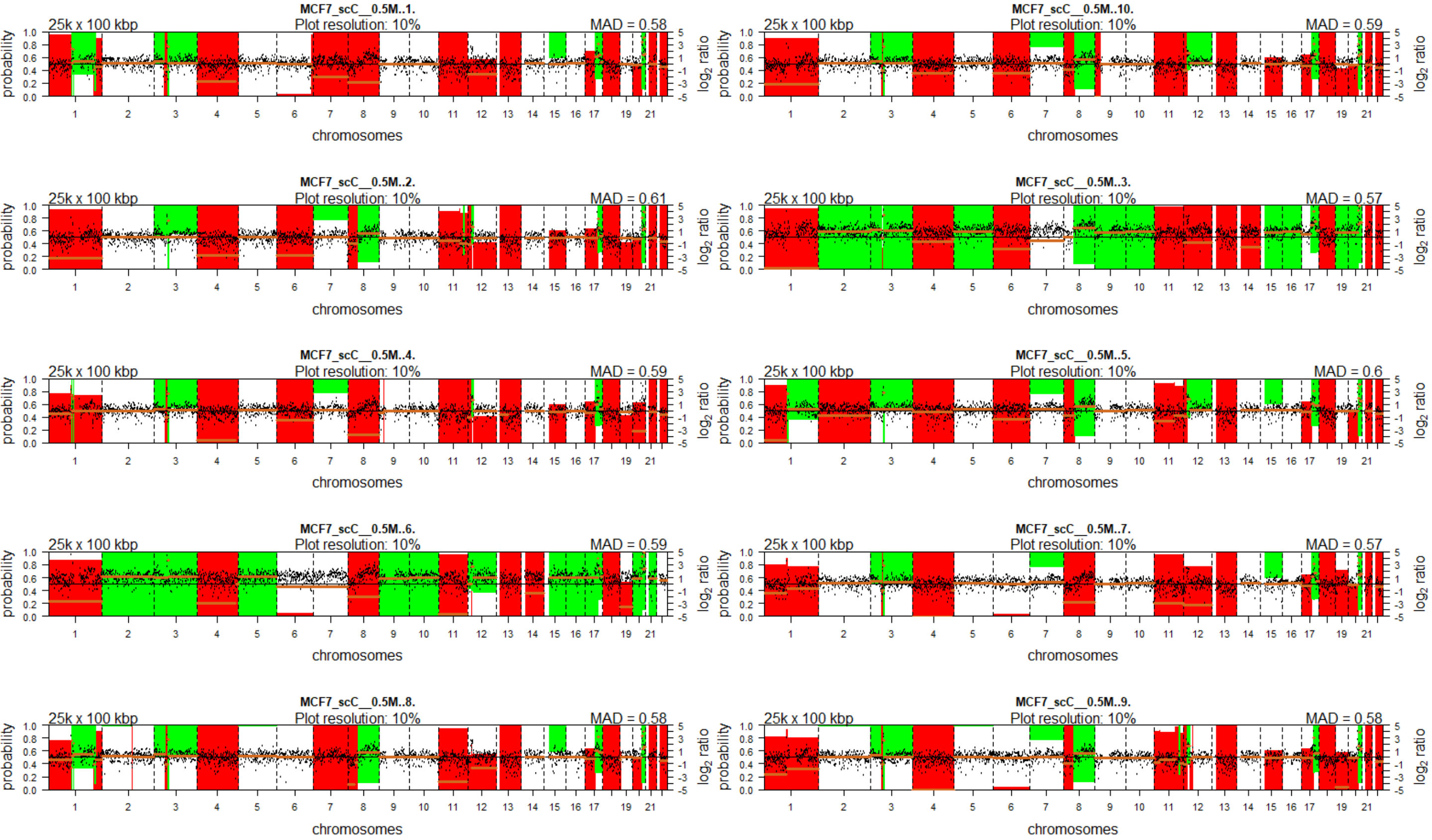
MCF7_scC__0.5M..1.
25k x 100 kbp
Plot resolution: 10%
MAD = 0.58
MCF7_scC__0.5M..10.
25k x 100 kbp
Plot resolution: 10%
MAD = 0.59
MCF7_scC__0.5M..2.
25k x 100 kbp
Plot resolution: 10%
MAD = 0.61
MCF7_scC__0.5M..3.
25k x 100 kbp
Plot resolution: 10%
MAD = 0.57
MCF7_scC__0.5M..4.
25k x 100 kbp
Plot resolution: 10%
MAD = 0.59
MCF7_scC__0.5M..5.
25k x 100 kbp
Plot resolution: 10%
MAD = 0.6
MCF7_scC__0.5M..6.
25k x 100 kbp
Plot resolution: 10%
MAD = 0.59
MCF7_scC__0.5M..7.
25k x 100 kbp
Plot resolution: 10%
MAD = 0.57
MCF7_scC__0.5M..8.
25k x 100 kbp
Plot resolution: 10%
MAD = 0.58
MCF7_scC__0.5M..9.
25k x 100 kbp
Plot resolution: 10%
MAD = 0.58
probability
log2 ratio
chromosomes
